# Structural basis for Pan3 binding to Pan2 and its function in mRNA recruitment and deadenylation

**DOI:** 10.15252/embj.201488373

**Published:** 2014-05-29

**Authors:** Jana Wolf, Eugene Valkov, Mark D Allen, Birthe Meineke, Yuliya Gordiyenko, Stephen H McLaughlin, Tayla M Olsen, Carol V Robinson, Mark Bycroft, Murray Stewart, Lori A Passmore

**Affiliations:** 1 Medical Research Council (MRC) Laboratory of Molecular BiologyCambridge, UK; 2 Chemistry Research Laboratory, University of OxfordOxford, UK

**Keywords:** gene expression, mRNA deadenylation, Pan2–Pan3, polyA tail, protein structure

## Abstract

The conserved eukaryotic Pan2–Pan3 deadenylation complex shortens cytoplasmic mRNA 3′ polyA tails to regulate mRNA stability. Although the exonuclease activity resides in Pan2, efficient deadenylation requires Pan3. The mechanistic role of Pan3 is unclear. Here, we show that Pan3 binds RNA directly both through its pseudokinase/C-terminal domain and via an N-terminal zinc finger that binds polyA RNA specifically. In contrast, isolated Pan2 is unable to bind RNA. Pan3 binds to the region of Pan2 that links its N-terminal WD40 domain to the C-terminal part that contains the exonuclease, with a 2:1 stoichiometry. The crystal structure of the Pan2 linker region bound to a Pan3 homodimer shows how the unusual structural asymmetry of the Pan3 dimer is used to form an extensive high-affinity interaction. This binding allows Pan3 to supply Pan2 with substrate polyA RNA, facilitating efficient mRNA deadenylation by the intact Pan2–Pan3 complex.

See also: **MT Stubbs & E Wahle** (July 2014)

## Introduction

In eukaryotic cells, mature mRNAs contain a 3′ polyadenosine (polyA) tail that is important for both mRNA stability and translation. The polyA tail is added co-transcriptionally to a length of approximately 70–90 nucleotides in *Saccharomyces cerevisiae* or approximately 200–250 nucleotides in humans (Zhao *et al*, 1999). Once mRNA is exported to the cytoplasm, the length of the polyA tail is regulated, which influences translation in some situations (Kapp & Lorsch, 2004; Goldstrohm & Wickens, 2008; Subtelny *et al*, 2014). For example, polyA tail length plays a key role in translational regulation of mRNAs in inflammation, neuronal processes, and early development (Weill *et al*, 2012). Deadenylation is also the first and rate-limiting step in cytoplasmic mRNA decay which involves one of two distinct pathways: (i) mRNA degradation by the exosome in a 3′–5′ direction or (ii) degradation from the 5′-end by decapping and the Xrn1 nuclease (Meyer *et al*, 2004; Parker & Song, 2004; Garneau *et al*, 2007).

PolyA tails are regulated at a mRNA-specific level (Lowell *et al*, 1992; Decker & Parker, 1993; Beilharz & Preiss, 2007; Lackner *et al*, 2007). Two conserved complexes carry out the majority of deadenylation in the cytoplasm: Pan2–Pan3 and Ccr4–Not (Wahle & Winkler, 2013). The catalytic activities are found in exonuclease-containing subunits Pan2 in Pan2–Pan3 and Ccr4 and Caf1/Pop2 in Ccr4–Not (Boeck *et al*, 1996; Tucker *et al*, 2001). Although these complexes appear to be at least partially functionally redundant, Pan2–Pan3 may be more efficient at initiating deadenylation, whereas Ccr4–Not may be more efficient at removing the final approximately 20–25 adenosines (Brown & Sachs, 1998; Tucker *et al*, 2001; Yamashita *et al*, 2005). This suggests that Pan2–Pan3 and Ccr4–Not could act sequentially. Severe phenotypes in yeast are only seen upon simultaneous deletion of both *PAN2* and *CCR4* (Tucker *et al*, 2001). Still, the molecular mechanisms of these complexes, and in particular the roles of non-enzymatic protein subunits, are unclear (Wiederhold & Passmore, 2010; Wolf & Passmore, 2014).

**Figure 1:**
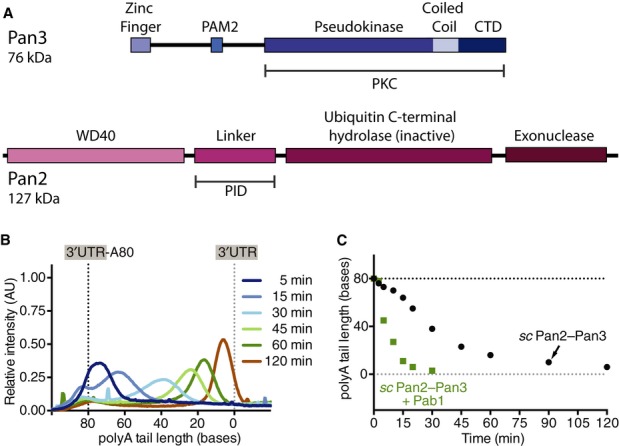
The deadenylation activity of the Pan2–Pan3 complex does not require PABP/Pab1 Schematic representation of Pan3 and Pan2 proteins. The N-terminus of Pan3 contains a zinc finger and a PABP interacting motif 2 (PAM2). The C-terminal part contains a pseudokinase, a coiled coil, and a C-terminal domain (CTD), which form a structural unit (PKC). Pan2 contains WD40, inactive ubiquitin C-terminal hydrolase (UCH), and exonuclease domains. A long linker region (Pan3 interacting domain, PID) connects the WD40 and UCH domains. Sizes are shown for *Saccharomyces cerevisiae* proteins and constructs used for crystallization are indicated below.Recombinant Pan2–Pan3 removes mRNA polyA tails. Deadenylation of a RNA substrate (*CYC1* 3′ UTR with A_80_ tail) by recombinant full-length *Saccharomyces cerevisiae* (*sc*) Pan2–Pan3 complex was analyzed by denaturing polyacrylamide gel electrophoresis (see Supplementary Fig S1). The lane profiles of various time points are shown here. The sizes of *CYC1* 3′ UTR RNA with (252 nt) and without (172 nt) an A_80_ tail are represented by black and gray dotted lines, respectively.Pab1 stimulates but is not required for Pan2–Pan3 deadenylation activity. Deadenylation assays using the same substrate as in (B) with *sc*Pan2–Pan3 in the absence (black) and presence (green) of the polyA-binding protein Pab1. The average length of the polyA tail is plotted for each time point where a single RNA band was visible on the gel (see Supplementary Fig S1). Dotted lines indicate the sizes of the *CYC1* 3′ UTR RNA with (black, 252 nt) and without (gray, 172 nt) a polyA_80_ tail.

Deadenylase complexes can be recruited to mRNA by binding RNA directly or indirectly through RNA binding proteins (Wahle & Winkler, 2013). The C-terminal domain of polyA-binding protein (PABP/Pab1) interacts with a short peptide motif (PABP interacting motif 2, PAM2) in Pan3 (Siddiqui *et al*, 2007) (Fig1A). PABP stimulates Pan2–Pan3 activity, both in yeast and human (Sachs & Deardorff, 1992; Uchida *et al*, 2004), and is assumed to be required for recruitment of Pan2–Pan3 to mRNA (Mangus *et al*, 2004b; Funakoshi *et al*, 2007; Siddiqui *et al*, 2007). Indeed, Pan2–Pan3 inefficiently removes the final 20–25 adenosines, the approximate length required to bind PABP (Baer & Kornberg, 1980; Sachs *et al*, 1987; Lowell *et al*, 1992). This suggests that Pan2–Pan3 activity stops or slows after the last PABP has been removed from the mRNA. A function of PABP to stimulate Pan2–Pan3 could play a role in the sequential model of deadenylation.

Pan3 has also been implicated in direct interactions with GW182 proteins in miRNA-mediated degradation, and with Dun1 kinase in regulation of Rad5 mRNA (Hammet *et al*, 2002; Braun *et al*, 2011; Chekulaeva *et al*, 2011; Fabian *et al*, 2011). In contrast, Pan2 is reported to interact only with Pan3 (Mangus *et al*, 2004a; Uchida *et al*, 2004). This led to the proposal that Pan3 acts as a regulator or adaptor within the complex, facilitating recruitment to mRNA via PABP (Uchida *et al*, 2004).

The mechanism of Pan2–Pan3 complex formation and substrate binding remain elusive. Recent work showed that Pan3 forms a homodimer in solution (Christie *et al*, 2013) raising the question of whether the Pan2–Pan3 complex contains one or two exonuclease subunits. Here, we use biochemical and structural approaches to define the mechanism of RNA recruitment by Pan2–Pan3 and demonstrate that the complex contains two Pan3 subunits and one Pan2 subunit. We show that Pan3 uses at least three different mechanisms to bind RNA, explaining its contribution to mRNA deadenylation. Further, we identify the region of Pan2 responsible for binding Pan3 and determine a crystal structure of their complex. This reveals an extensive interface underscoring the importance of a stable interaction between these two proteins to allow Pan3 to supply Pan2 with substrate RNA for efficient mRNA deadenylation.

## Results

### The deadenylation activity of Pan2–Pan3 does not require Pab1

To investigate the molecular mechanism of mRNA deadenylation by Pan2–Pan3, we purified recombinant *S. cerevisiae* Pan2–Pan3 complex (*sc*Pan2–Pan3) and polyA-binding protein Pab1 (Supplementary Fig S1A). We tested the ability of *sc*Pan2–Pan3 to remove a 3′ polyA tail from an *in vitro* transcribed model mRNA comprising the 3′ untranslated region (UTR) from the *CYC1* gene followed by 80 adenosines. The *CYC1* 3′ UTR has been used extensively to study the addition of polyA tails during mRNA processing (Viphakone *et al*, 2008). In our assays, the length of the RNA substrate was monitored by gel electrophoresis. We observed robust activity of *sc*Pan2–Pan3, which removed the polyA_80_ tail, even in the absence of Pab1 (Fig1B and C and Supplementary Fig S1). In contrast, Pan2–Pan3 complex containing a catalytic mutation in Pan2 (E912A) did not have nuclease activity (Supplementary Fig S2). Thus, the purified Pan2–Pan3 complex is an active deadenylase *in vitro* and appears to have an intrinsic ability to bind polyA RNA.

Addition of Pab1 to *sc*Pan2-Pan3 stimulated the rate of the reaction by approximately fourfold (Fig1C and Supplementary Figs S1 and S2). Nuclease activity continued beyond the polyA tail, presumably due to binding of Pab1 to A-rich regions of the 3′ UTR. In contrast, in the absence of Pab1, *sc*Pan2–Pan3 did not degrade the 3′ UTR region of the substrate and stopped after the 80 adenosines had been removed (Fig1C and Supplementary Fig S1).

In the absence of Pab1, it appears that removal of the final approximately 25 adenosines occurs at a slower rate than removal of the first 50 adenosines (Fig1C and Supplementary Fig S1C). This is similar to a previous study where removal of the last 10–25 adenosines by Pan2–Pan3 was inefficient (Lowell *et al*, 1992). Our results suggest that inefficient removal of short polyA tails is not necessarily due to dissociation of the last Pab1/PABP and the polyA specificity is not solely determined by polyA-binding proteins. Instead, this behavior is determined primarily by the interaction of Pan2–Pan3 with mRNA itself.

### The Pan3 CCCH zinc finger binds polyA RNA

Since Pab1/PABP was not necessary for Pan2–Pan3 to deadenylate RNA in the *in vitro* assay, we analyzed the role of either Pan3 or Pan2 alone in RNA binding. As illustrated in Fig1A, Pan3 contains a zinc finger at its N-terminus, a pseudokinase domain (PK), a coiled coil and a highly conserved C-terminal domain (CTD) (Brown *et al*, 1996; Christie *et al*, 2013; Wolf & Passmore, 2014). Pan2 contains three functional domains: an N-terminal WD40 domain, an inactive ubiquitin C-terminal hydrolase (UCH) domain and a C-terminal exonuclease (Fig1A) (Boeck *et al*, 1996; Wolf & Passmore, 2014). A linker region separates the WD40 and UCH domains. Although zinc fingers often act as interaction modules with DNA, RNA or protein (Font & Mackay, 2010), none of the domains in either Pan3 or Pan2 have previously been shown to bind directly to RNA.

**Figure 2:**
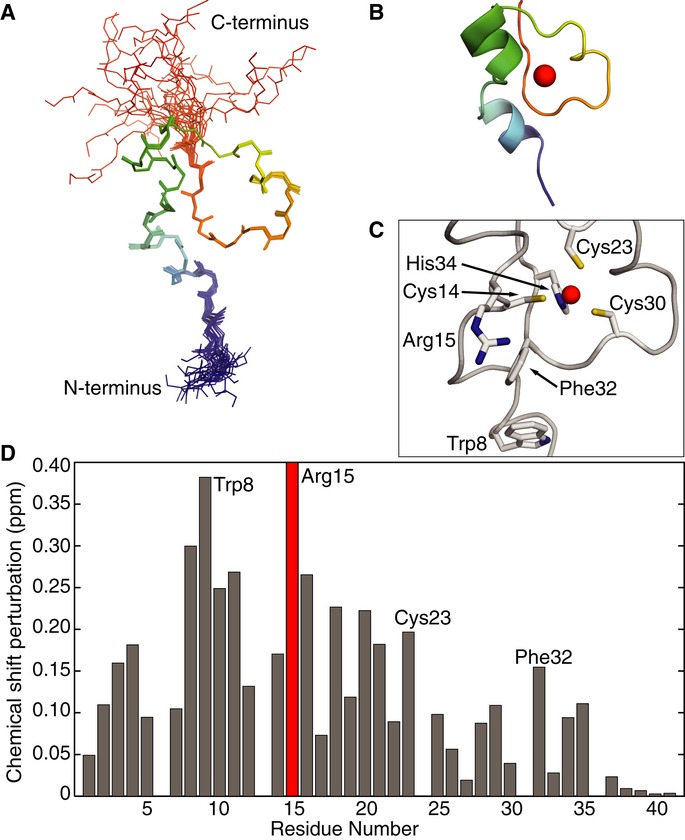
The zinc finger domain of *sc*Pan3 binds polyA RNA Overlay of the 20 best NMR models of the CCCH zinc finger of *Saccharomyces cerevisiae* Pan3 (residues 1–41) showing the protein backbone colored in rainbow from N- to C-terminus.Cartoon representation of the zinc finger of *sc*Pan3 with a modeled zinc ion (red sphere). The disordered termini have been omitted.Close-up of the zinc finger of *sc*Pan3 (ribbon) showing functional residues in stick representation. The Zn atom (red sphere) is coordinated to His34 and the three cysteines.The *sc*Pan3 zinc finger binds polyA RNA. Plot of the magnitude of chemical shift perturbation of each residue, calculated from ^15^N-^1^H HSQC spectra of 150 μM *sc*Pan3 zinc finger after addition of 150 μM 15-mer polyA RNA. Many of the zinc finger residues experience large changes in their chemical environment in the presence of polyA RNA. Residues discussed in the text are labeled. The peak for Arg15 (red) disappears upon RNA binding. (Also see Supplementary Fig S4).

We determined the solution NMR structure of the zinc finger in *sc*Pan3 (residues 1–41) and used this to investigate its role in binding RNA. Complete ^1^H/^13^C/^15^N assignments and structure determination were carried out using conventional methods (Supplementary Table S1). The N-terminus of *sc*Pan3 has a compact CCCH-type zinc finger with a well-defined core, held in place by the zinc ion (Fig2A–C). It also has an N-terminal extension that is largely ordered. The zinc-coordinating residues Cys14, Cys23, Cys30, and His34 (CCCH) of *sc*Pan3 are universally conserved in Pan3 zinc fingers (Supplementary Fig S3A). The overall structure is similar to the CCCH zinc fingers in MBNL1 (muscleblind-like protein), TIS11d (Tristetraprolin family), and Nab2 (Hudson *et al*, 2004; Teplova & Patel, 2008; Brockmann *et al*, 2012; Kuhlmann *et al*, 2014) (Supplementary Fig S3). The TIS11d zinc finger is strikingly similar, including an ordered N-terminal extension, a short α-helix between the first two zinc-coordinating cysteines and a 3_10_ helix between the second and third cysteines. Interestingly, MBNL1, TIS11d and Nab2 all bind RNA.

To investigate whether the *sc*Pan3 zinc finger can also bind RNA, we performed chemical shift perturbation experiments to measure changes in its environment in the presence of polyA_15_, C_15_, G_15_, or U_15_ RNA. Large shifts of the peaks of many zinc finger residues were observed in the presence of polyA, whereas polyG generated small changes and polyC or polyU generated negligible changes (Fig2D and Supplementary Fig S4). By titrating the amount of RNA in the NMR experiment, we estimate the binding affinity of this zinc finger to the polyA_15_ RNA to be 60–100 μM (Supplementary Fig S4). These data indicate that the zinc finger of *sc*Pan3 binds polyA RNA in preference to other polyribonucleotides.

### Pan3 pseudokinase/CTD interacts directly with RNA

**Figure 3:**
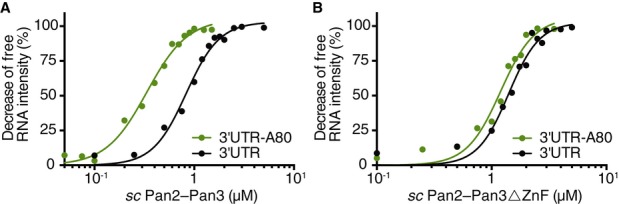
The zinc finger domain of *sc*Pan3 increases the affinity of the Pan2–Pan3 complex to polyA RNA A, B Electrophoretic mobility shift assay (EMSA) using the *CYC1* 3′ UTR RNA with (green) or without (black) a polyA_80_ tail. RNA was incubated with (A) *sc*Pan2–Pan3 or (B) *sc*Pan2–Pan3 with a deletion of the Pan3 zinc finger (*sc*Pan2–Pan3ΔZnF). Both complexes contain an active site mutation in *sc*Pan2 (E912A). Binding was analyzed by native polyacrylamide gel electrophoresis, and the decrease in the intensity of free RNA was quantified and plotted against protein concentration for a representative experiment. Also see Supplementary Fig S5A and B.

To assess whether the zinc finger is the only component in the Pan2–Pan3 complex that binds directly to RNA, we deleted it from *sc*Pan3 and investigated the ability of the truncated *sc*Pan2–Pan3 complex to bind RNA. We used an active site mutant in *sc*Pan2 (E912A) to prevent degradation of the substrate *CYC1* 3′ UTR model RNA (with or without an A_80_ tail) in electrophoretic mobility shift assays (EMSAs). The EMSA experiments in Fig3 and Supplementary Fig S5A and B show that wild-type *sc*Pan2–Pan3 and *sc*Pan2–Pan3 lacking the zinc finger both bind *CYC1* RNA, irrespective of whether it has a polyA tail. Wild-type *sc*Pan2–Pan3 has a slightly higher affinity for polyadenylated (A_80_) RNA compared to the non-adenylated 3′ UTR (Fig3A). In contrast, *sc*Pan2–Pan3 lacking the zinc finger domain showed the same binding affinity for *CYC1* RNA with and without polyA tail (Fig3B). Together, these data show that although the *sc*Pan3 zinc finger binds RNA and may convey polyA specificity, other parts of Pan2–Pan3 contribute to RNA binding. In agreement with this, *sc*Pan2–Pan3 lacking a zinc finger has a slightly reduced rate of deadenylation compared to wild-type complex (Supplementary Fig S5C).

**Figure 4:**
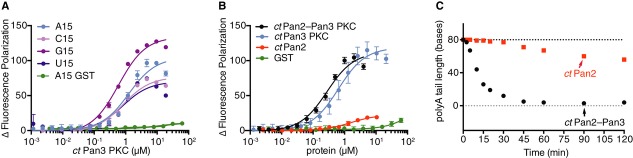
Pan3 functions within the Pan2–Pan3 complex to bind RNA Pan3 pseudokinase/C-terminal domain (PKC) from *Chaetomium thermophilum* (*ct*) binds RNA but has no preference for polyA. The plot shows a comparison of the change in the fluorescence polarization signal of 20 nM 5′ Cy3-labeled polyA, polyC, polyG, and polyU RNA upon addition of *ct*Pan3 pseudokinase/C-terminal domain (PKC) or purified GST, for a representative experiment. Error bars are the standard deviations of triplicate measurements from one experiment. The *K*_d_s for RNA binding are: 2.1 ± 1.4 μM (A15); 2.2 ± 1.7 μM (C15); 0.55 ± 0.05 μM (G15); and 1.6 ± 0.6 μM (U15) (average of three independent experiments, errors are the standard deviations).Isolated *ct*Pan2 has a low affinity for RNA compared with *ct*Pan3 PKC. The change in the fluorescence polarization signal of 20 nM 5′ Cy3-labeled polyA RNA upon addition of *ct*Pan2 (red), *ct*Pan3 PKC (blue), *ct*Pan2–Pan3 PKC (black), or purified GST (green) is shown as the average of three independent experiments. Error bars are standard deviations. The *K*_d_s for A25 RNA binding are: 0.62 ± 0.06 μM (*ct*Pan3 PKC) and 0.27 ± 0.02 μM (*ct*Pan2–Pan3 PKC) (average of three independent experiments, errors are the standard deviations).Intact *ct*Pan2–Pan3 complex (black) has much higher activity than isolated *ct*Pan2 (red). Deadenylation assays using *CYC1* 3′ UTR with A_80_ tail (Supplementary Fig S1). The average length of the polyA tail is plotted for each time point. Dotted lines indicate the sizes of the *CYC1* 3′ UTR RNA with (black, 252 nt) and without (gray, 172 nt) a polyA_80_ tail.

To evaluate whether other domains of Pan3 contribute to RNA binding, we used a short synthetic RNA that could be more readily quantitated. We measured the changes in the fluorescence polarization signal of 5′ Cy3-labeled polyA_15_ RNA upon addition of a truncated Pan3 protein containing the pseudokinase and C-terminal domains (PKC). Separately expressed Pan2 and Pan3 proteins from budding yeast had a tendency to aggregate in isolation and so we instead used proteins from the thermophilic fungus *Chaetomium thermophilum* (*ct*) for these experiments (Supplementary Fig S1A). We observed an increase in polarization of the labeled RNA showing that *ct*Pan3 PKC can bind polyA (Fig4A). Similar binding affinities could be observed when polyC_15_ or U_15_ RNA was used, indicating that the interaction of this region of *ct*Pan3 with RNA is not polyA specific (Fig4A). The interaction with polyG_15_ appeared tighter but interpretation of this result is complicated by an increased magnitude of fluorescence polarization, possibly due to the well-known potential of polyG to form G-quadruplexes (Joachimi *et al*, 2009). Thus, the PKC region of *ct*Pan3 also binds RNA but is not specific for polyA.

### Pan2 does not bind RNA and requires Pan3 for efficient deadenylation

To determine whether Pan2 also binds RNA, we again measured the changes in fluorescence polarization of a labeled polyA RNA after incubation with protein. Changes in the polarization signal of the RNA were substantially reduced in the presence of catalytically inactive *ct*Pan2 compared with *ct*Pan3 PKC (Fig4B). As a control, GST (which is not expected to interact with RNA) did not substantially influence the fluorescence polarization signal (Fig4B). Thus, the interaction of *ct*Pan2 with polyA RNA is much weaker than the interaction of *ct*Pan3 PKC with the same RNA. When we tested the interaction of the *ct*Pan2–Pan3 PKC complex with polyA RNA, the binding was comparable to *ct*Pan3 PKC alone (Fig4B).

The data in Fig4B suggest that interaction of *ct*Pan2 with polyA RNA is negligible. Consequently, we predicted that the activity of isolated Pan2 would be very weak. To test this, we used isolated *ct*Pan2 in the *in vitro* deadenylation assay described above. As shown in Fig4C and Supplementary Fig S1, *ct*Pan2 alone has greatly reduced activity compared to the Pan2–Pan3 complex and only removes approximately 30 As in 2 h. In comparison, full-length *ct*Pan2–Pan3 removes almost the entire polyA_80_ tail in 30 min. Like the *Saccharomyces cerevisiae* proteins, the complex from *Chaetomium* exhibits robust activity in the absence of Pab1, whereas *ct*Pan2–Pan3 containing a catalytic mutation in Pan2 (E899A) is not active (Supplementary Fig S2C). These results show that Pan2 does not deadenylate mRNA efficiently in the absence of Pan3.

### One Pan2 linker region mediates binding to a Pan3 homodimer

The experiments described above highlight the importance of association of Pan2 and Pan3 into a complex for their function in mRNA deadenylation, so we characterized how these two proteins interact at the molecular level in greater detail. Yeast-two-hybrid and mutational studies suggest that the CTD of Pan3 is involved in the interaction with Pan2 (Mangus *et al*, 2004a; Christie *et al*, 2013). Furthermore, the WD40 or exonuclease domains of Pan2 can be deleted without losing the interaction with Pan3 (Mangus *et al*, 2004a; Uchida *et al*, 2004).

**Figure 5:**
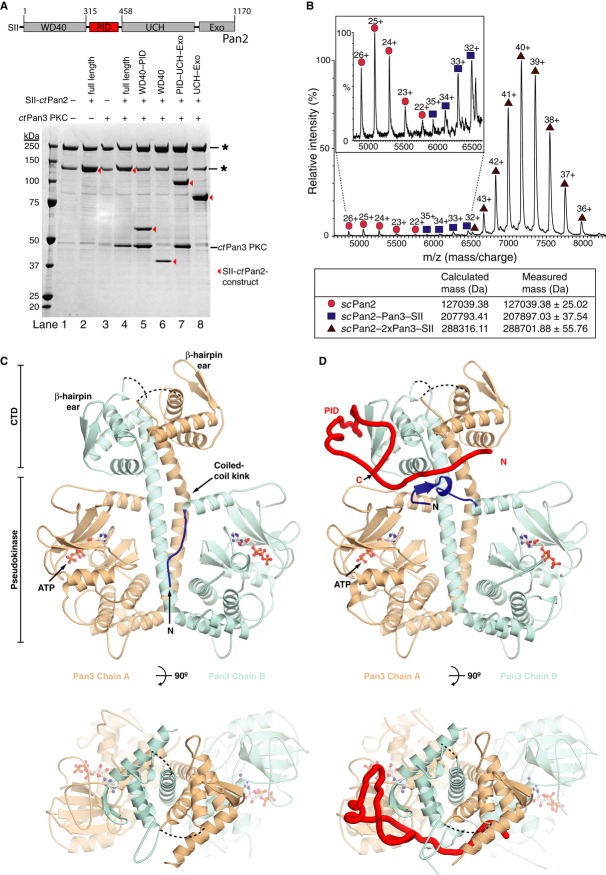
The Pan2–Pan3 complex forms a heterotrimer A *ct*Pan3 interacts with the linker region (Pan3 interacting domain, PID) of *ct*Pan2. Various truncations of StrepII (SII)-tagged *ct*Pan2 (full length, 1–1170; WD40-PID, 1–458; WD40, 1–315; PID-UCH-Exo, 316–1170; UCH-Exo, 459–1170) were co-expressed with *ct*Pan3 pseudokinase/C-terminal domain (PKC, 205–640) in *Saccharomyces cerevisiae*, and pull-downs were performed using StrepTactin beads. *ct*Pan2 proteins are indicated by a red arrowhead. Asterisks indicate contaminating bands that bind to StrepTactin beads. B Intact Pan2–Pan3 has a stoichiometry of 1:2. NanoESI-MS spectrum of recombinant *sc*Pan2-Pan3, showing the major peak series (< 95%) of a heterotrimeric complex of one Pan2 and two Pan3 molecules at 6,500–8,000 m/z (dark red triangles). C, D The *ct*Pan2 linker region wraps around a homodimer of *ct*Pan3. Crystal structures of (C) *ct*Pan3 PKC homodimer and (D) *ct*Pan3 PKC bound to the Pan3 interacting domain (PID) of *ct*Pan2. Pan3 chains A and B are shown in wheat and cyan, respectively, and *ct*Pan2 PID is shown in red. The N-terminal residues of Pan3 chain B that change conformation on Pan2 binding are shown in dark blue. ATP is shown in ball and stick representation. Dashed lines connect loops not visible in the electron density. The two views are related by a rotation of 90° around the horizontal axis.

To map the Pan3 binding site on Pan2, we over-expressed a series of StrepII-tagged *ct*Pan2 truncations together with *ct*Pan3 PKC and used pull-down experiments to test for their interaction. Removal of either the C-terminal UCH and exonuclease domains or the N-terminal WD40 domain did not impair the ability of *ct*Pan2 to bind *ct*Pan3 PKC (Fig5A, lanes 5 and 7). However, additional removal of the linker that connects the WD40 and UCH domains resulted in loss of the interaction with *ct*Pan3 PKC (Fig5A, lanes 6 and 8). Together, these data indicate that the linker region of Pan2 (residues 316–458 for *ct*Pan2) mediates the interaction with Pan3. Hence, we refer to this region as the ‘Pan3 interaction domain’ (PID).

A recent crystal structure of Pan3 revealed that it forms a homodimer (Christie *et al*, 2013). Accompanying biochemical data suggested that mutants that were unable to dimerize could still bind Pan2. These data indicated that a Pan3 dimer might bind two copies of Pan2 with an overall stoichiometry of 2:2. To establish the stoichiometry of the Pan2–Pan3 complex, we used non-covalent nanoelectrospray ionization mass spectrometry (nanoESI-MS). This technique allows the determination of composition and stoichiometry of native protein complexes (Hernández & Robinson, 2007). NanoESI-MS of *sc*Pan2–Pan3 revealed that the most prominent species (> 95%) corresponds to a heterotrimeric complex containing one *sc*Pan2 and two *sc*Pan3 molecules (Fig5B). These data are consistent with formation of a Pan2–Pan3 1:2 heterotrimer in solution.

### Structural basis of *Chaetomium thermophilum* Pan2–Pan3 complex formation

To understand the basis of subunit association in the Pan2–Pan3 complex, we determined the crystal structure of the pseudokinase/C-terminal domains of Pan3 alone and when in complex with the Pan3 interaction domain of Pan2. Proteins obtained from *C. thermophilum* generated crystals that diffracted to 2.4 Å resolution, substantially higher than that obtained from *Drosophila melanogaster* or *Neurospora crassa* Pan3 crystals (Christie *et al*, 2013), and so these were used for structural characterization. We obtained crystals of Se-Met-labeled *ct*Pan3 PKC (residues 205–640) with *P1* symmetry and four Pan3 homodimers in the asymmetric unit that were solved using single-wavelength anomalous dispersion (SAD) methods (Supplementary Table S2).

The *ct*Pan3 structure consists of an N-terminal pseudokinase domain (residues 242–497) to which MgATP is bound, a central asymmetric coiled-coil domain (residues 499–538) and a C-terminal domain (residues 555–640) (Fig5C). Although ATP was bound to both pseudokinase domains, ATP was not added to crystallization conditions and is present in all copies in the asymmetric unit of the crystal. The pseudokinase lacks almost all catalytic residues and is predicted to be inactive (Christie *et al*, 2013). Two distinctive beta-hairpin ‘ears’ protrude from each C-terminal domain. A kink is formed in the coiled-coil region as a result of one of the CTDs (chain B) interacting with the pseudokinase from the other chain, introducing asymmetry into the Pan3 dimer. The pseudokinase and CTD domains of *C. thermophilum* Pan3 are similar to the recently reported structures for *Drosophila* (RMSDs of 1.13 Å and 0.87 Å, respectively) and *Neurospora* (RMSDs of 1.24 Å and 0.93 Å, respectively) proteins (Christie *et al*, 2013), indicating high structural conservation of Pan3 (Supplementary Fig S6A and B). The principal difference between these structures is that the kink in the coiled coil of *ct*Pan3 is more pronounced, resulting in twists of approximately 28° and 31°, respectively, of the CTD relative to the pseudokinase domain.

We also crystallized *ct*Pan3 PKC bound to the PID of *ct*Pan2. The best diffracting crystals were obtained with a construct that lacks the first 27 residues of the PID region, *ct*Pan2^343–458^, had *P2*_*1*_ symmetry and diffracted to 2.6 Å resolution (Supplementary Table S3) with two copies of the complex in the asymmetric unit. Molecular replacement using the structure of *ct*Pan3 PKC showed clear difference density corresponding to a single *ct*Pan2 chain (comprising residues 355–406) on each *ct*Pan3 dimer. The overall structure of the *ct*Pan3 dimer in the complex closely resembled that obtained for *ct*Pan3 alone (C_α_ RMSD 0.94 Å), although there was a slight (˜6°) rotation of the C-terminal domain relative to the coiled-coil and pseudokinase domain (Fig5C and D and Supplementary Fig S6C). ATP was bound to the pseudokinase domains. The major difference between free and Pan2-associated *ct*Pan3 was a substantial rotation of the N-terminus of chain B (residues 207–224) by approximately 90° toward the Pan3 CTD (Fig5C and D). This region had been removed from the *Neurospora* and *Drosophila* constructs used in previous crystallization studies (Christie *et al*, 2013).

**Figure 6:**
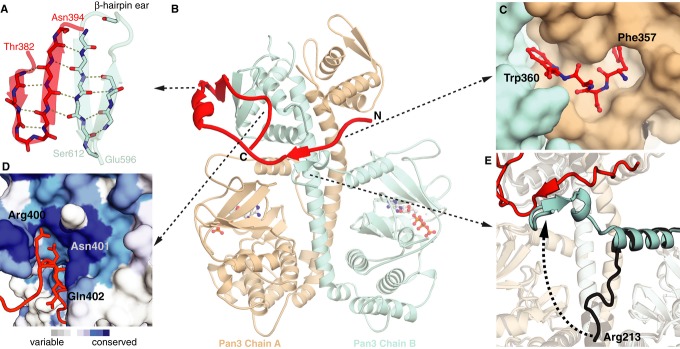
Pan2 exploits the asymmetry and forms an extensive interface with the Pan3 dimer Structural details of the complex of *ct*Pan3 PKC bound to the Pan3 interacting domain (PID) of *ct*Pan2. *ct*Pan2 is shown in red, and *ct*Pan3 PKC chains A and B are depicted in wheat and cyan, respectively. Close-up view of the four-stranded intermolecular antiparallel β-sheet formed between a β-hairpin in *ct*Pan2 (residues 384–393) and a β-hairpin ear in *ct*Pan3 chain B (residues 597–612).Overall structure of the *ct*Pan3 PKC dimer bound to *ct*Pan2 PID (cartoon). ATP is in ball and stick representation.The N-terminal region of *ct*Pan2 PID interacts with the *ct*Pan3 dimer by inserting aromatic residues Phe357 and Trp360 (stick representation) into two pockets formed between the coiled coil and the CTD of *ct*Pan3 chain A.Surface representation of the CTD of *ct*Pan3 chain B colored by sequence conservation. *ct*Pan2 PID (residues 400–402) binds into a conserved groove in the CTD.Superposition of c*t*Pan3 from the structures of isolated *ct*Pan3 (black) and bound to *ct*Pan2 PID (Pan3 in cyan and wheat, Pan2 in red) in cartoon representation. The N-terminus of chain B of the *ct*Pan3 dimer rotates by approximately 90° upon binding of *ct*Pan2 to form an antiparallel β-sheet with *ct*Pan2.

The PID of *ct*Pan2 exploits the asymmetry of the *ct*Pan3 dimer by wrapping around it at the interface between the pseudokinase and C-terminal domain (Fig6). The N-terminus of the *ct*Pan2 PID (residues 355–360) interacts with chain A of the *ct*Pan3 dimer. In particular, *ct*Pan2 Phe357 and Trp360 are inserted into a pocket of *ct*Pan3 formed by the C-terminal domain of chain A and the coiled coil (Fig6C). Phe357 and Trp360 are conserved hydrophobic residues among Pan2 orthologs (Supplementary Fig S6D).

The PID of *ct*Pan2 next passes over the coiled coil and interacts with the N-terminus of *ct*Pan3 chain B (Gln212–Ala214) through an intermolecular antiparallel β-sheet (Fig6E). This new β-sheet is located between the pseudokinase domain of chain A and the CTD of chain B. The PID continues around Pan3 to form extensive interactions with the CTD of chain B that include an extended 4-strand antiparallel β-sheet between a β-hairpin of the *ct*Pan2-PID (residues 384–393) and a β-hairpin ‘ear’ in *ct*Pan3 (residues 597–612) (Fig6A). Finally, residues 399–403 of the PID of *ct*Pan2 bind in a highly conserved groove formed by residues 599–603 and 562–573 in the CTD of chain B of the Pan3 dimer (Fig6D). Interestingly, binding of *ct*Pan2 to *ct*Pan3 is mediated mainly by interactions between main chain atoms, not side chains.

### The extensive Pan2–Pan3 interface generates high affinity

Collectively, the interface between the PID of *ct*Pan2 and the *ct*Pan3 PKC dimer buries a surface area of approximately 2,230 Å^2^, and the *ct*Pan3 PKC dimer interface buries approximately 3,740 Å^2^. The extensive surface area buried between these two proteins, together with the hydrogen bonding associated with new secondary structural elements formed on binding, is consistent with a tight association. We characterized the interaction of *ct*Pan3 PKC with *ct*Pan2 PID thermodynamically and kinetically and found a *K*_d_ of 10 nM using surface plasmon resonance and a *K*_d_ approximately 50 nM with isothermal titration calorimetry, though the necessity to use low protein concentrations for this range of affinities in the latter places a limit on the accuracy of this value (Supplementary Fig S7A–C). A large favorable change in enthalpy (ΔH ˜−30 kcal/mol) was observed during the interaction that was opposed by an unfavorable change in the entropy of the system (−TΔS ˜20 kcal/mol). These values reflect the extensive interactions between *ct*Pan2 and *ct*Pan3 and a potential loss in conformational flexibility upon binding. The stoichiometry of interaction confirmed that only a single Pan2 chain was bound to each Pan3 dimer in solution.

### The Pan2 PID observed in the crystal structure is important for Pan3 binding in solution

As we observed only 52 (residues 355–406) of the 142 residues of the Pan2 linker that connects the WD40 and UCH domains in the electron density, we analyzed the contributions made by different regions of the PID to the interaction. Pull-down assays using *ct*Pan3 PKC and N- or C-terminal truncations of *ct*Pan2 (Fig5A and Supplementary Fig S7D and E) suggest that the major Pan3 binding region on *ct*Pan2 includes the segment seen in the crystal structure. Since amino acids 1–342 and 1–352 also bind weakly to *ct*Pan3, there may be additional contributions from these N-terminal residues. Notably, no region is absolutely required for binding, consistent with the extensive interface observed in the crystal structure.

**Figure 7:**
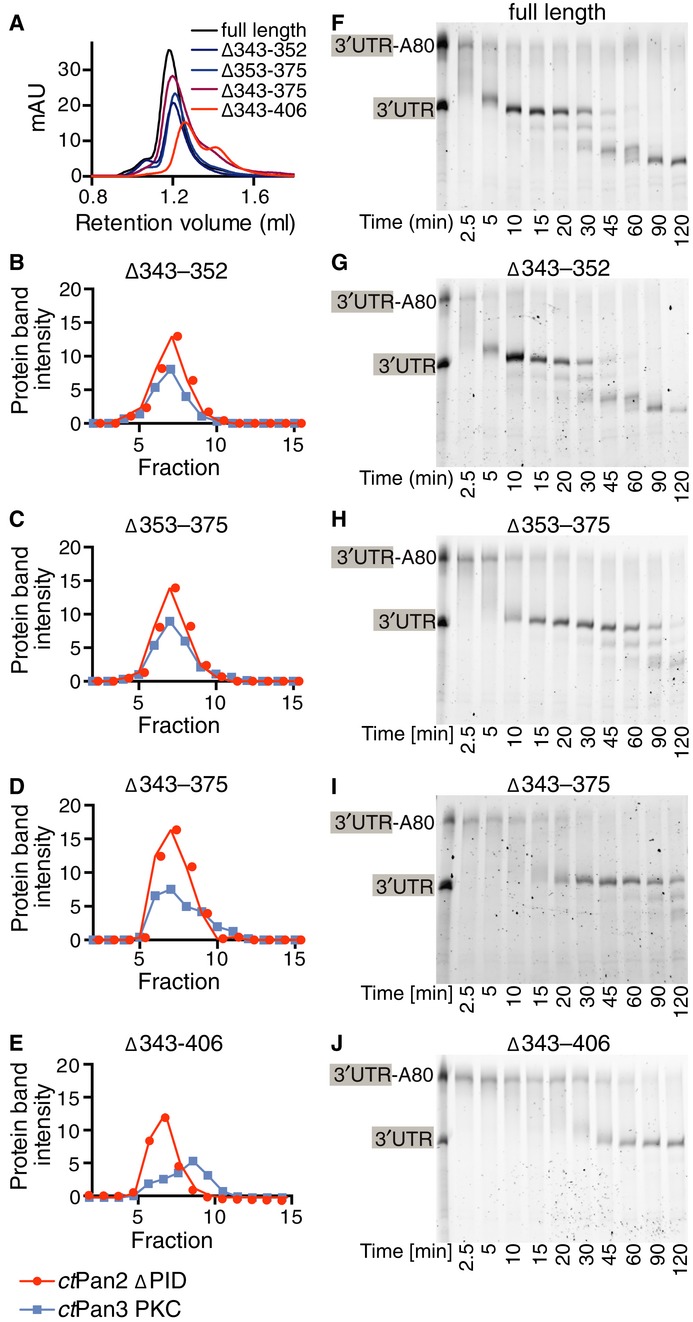
The *ct*Pan2 PID observed in the crystal structure interacts with *ct*Pan3 PKC in solution A–E Deletion of 63 residues of the PID is required to disrupt the interaction between *ct*Pan2 and *ct*Pan3 *in vitro*. Separately purified *ct*Pan2 PID deletion constructs and *ct*Pan3 PKC were mixed, and binding was analyzed by size exclusion chromatography. (A) Elution profiles measuring A_280 nm_ are shown for *ct*Pan3 PKC with full-length *ct*Pan2 (black) and containing the following deletions in the PID: Δ343–352 (dark blue), Δ353–375 (light blue), Δ343–375 (dark red), and Δ343–406 (red). (B–E) Fractions of the size exclusion chromatography runs from (A) were analyzed by SDS–PAGE, and intensities of individual protein bands were quantified and plotted against fraction number. *ct*Pan2 and *ct*Pan3 PKC are depicted in red and blue, respectively. F–J A similar pattern was observed when the activity of the deletion constructs was assessed using deadenylation of an RNA substrate (*CYC1* 3′ UTR with A_80_ tail) by *ct*Pan3 PKC mixed with (F) full-length *ct*Pan2 or (G–J) PID deletion mutants. Time points were analyzed by denaturing polyacrylamide gel electrophoresis. The first lane shows *CYC1* 3′ UTR with and without A_80_ tail as a marker. Although the Δ353–375 and Δ343–375 constructs showed a slight decrease in deadenylation rate, a major decrease was only observed with the Δ343–406 construct that also dissociated during size exclusion chromatography (E).

We further assayed the ability of *ct*Pan2 mutants to bind *ct*Pan3 PKC by mixing separately purified proteins and analyzing whether they co-elute on size exclusion chromatography. Because of the extensive nature of the interaction interface, point mutations even of deeply buried residues such as Phe357 and Trp360 were not able to impair the interaction (Supplementary Fig S7F). We therefore assayed the effect of internal deletions of the PID in the context of full-length *ct*Pan2 (Fig7A–E). We assessed the contributions of: (i) residues 343–352 for which electron density was not visible in our structure, (ii) the N-terminal region of the PID visible in our structure (residues 353–375), and (iii) the C-terminal half of the PID observed in the crystal structure, residues 376–406 (Supplementary Fig S6D). Only simultaneous deletion of all three regions (Δ343–406) abrogated the co-elution of *ct*Pan2 and *ct*Pan3 PKC on size exclusion chromatography, resulting in the appearance of separate peaks (Fig7A and 7E). Consistent with these data, when we tested the deadenylation activity of *ct*Pan2–Pan3 PKC complexes formed with PID deletion mutants, larger deletions resulted in decreased activity (Fig7F–J). For example, full-length *ct*Pan2–Pan3 PKC deadenylated all the RNA after approximately 10 min (Fig7F), whereas the Δ343–406 mutant required approximately 90 min for full deadenylation (Fig7J). Because the Δ343–406 mutant retained significant activity compared to isolated *ct*Pan2, there may be additional sites of Pan2–Pan3 interaction, either within the PID (such as residues 316–342, Fig5A and S7D) or outside the PID.

In summary, we found that it was difficult to disrupt the Pan2–Pan3 interaction by deletion of substantial regions of *ct*Pan2 PID and show that a core region (residues 343–406) makes a major contribution to the association of *ct*Pan2 and *ct*Pan3. These results are consistent with the extensive interaction interface observed in the crystal structure of the complex and the low nanomolar binding affinity of *ct*Pan2 to *ct*Pan3.

## Discussion

Although Pan2 provides the catalytic activity of the Pan2–Pan3 deadenylation complex and defects in Pan3 lead to loss of Pan2 function (Brown *et al*, 1996; Boeck *et al*, 1998; Uchida *et al*, 2004), the mechanism by which Pan3 facilitates deadenylation was unclear. Together, our data suggest that the role of Pan3 in the Pan2–Pan3 complex is to supply Pan2 with substrate polyA RNA. We show that the reduced deadenylase activity of isolated Pan2 is likely due to an inability to bind RNA. In the complex, Pan3 functions to bind RNA through at least three different mechanisms, facilitating recruitment of Pan2 to the polyA tail. The Pan3 homodimer binds a single Pan2 chain, and the crystal structure of *ct*Pan3 bound to the interacting linker region of *ct*Pan2 (residues 355–406) shows an extensive interface between the two subunits consistent with the integral role of Pan3 in the overall function of the complex.

### Pan3 supplies Pan2 with substrate RNA through complementary mechanisms

Although an interaction between the PAM2 motif of Pan3 and PABP/Pab1 can mediate indirect recruitment of Pan2–Pan3 to the polyA tail (Siddiqui *et al*, 2007), our data demonstrate that Pan3 can function through at least two additional complementary mechanisms to bind RNA directly. First, the N-terminal zinc finger of Pan3 conveys sequence specificity by binding to polyA RNA preferentially over other polyribonucleotides (Supplementary Fig S4). The NMR solution structure of the *sc*Pan3 zinc finger (Fig2) revealed a similarity to other zinc fingers that bind RNA (Hudson *et al*, 2004; Teplova & Patel, 2008; Kuhlmann *et al*, 2014). Each of these other proteins contains tandem zinc fingers within the same polypeptide chain (four in MBNL1, two in TIS11d, and seven in Nab2), probably because the affinity of a single finger is insufficient to provide binding specificity. In Pan2–Pan3, there are two zinc fingers (one in each Pan3 chain) that may contribute to recognition in an analogous manner.

Second, the highly conserved Pan3 pseudokinase/C-terminal domain also binds RNA but in a manner that is not specific for polyA (Fig4). This region has a theoretical pI of approximately 8.8, and examination of the surface potential reveals a substantial positive charge (Supplementary Fig S8) that could mediate electrostatic interactions with negatively charged RNA backbone phosphates. In contrast, the ability of isolated Pan2 to bind RNA is negligible, and it has very low deadenylation activity in the absence of Pan3 (Fig4).

The importance of correct substrate selection is highlighted by the three distinct ways that Pan3 can use to recognize polyA RNA. Each individual interaction is weak: *K*_d_ of approximately 40–150 μM for the PAM2:PABP interaction (Siddiqui *et al*, 2007), apparent *K*_d_ of approximately 60–100 μM for the zinc finger:polyA interaction (Supplementary Fig S4), and *K*_d_ of approximately 0.5–2 μM for the PKC:RNA interaction (Fig4A). Because Pan3 is a dimer, there are two zinc fingers and two PAM2 motifs in each Pan2–Pan3 complex. Consequently, avidity effects due to these interactions acting in concert likely increase the overall affinity substantially, thereby facilitating recruitment to mRNA.

### The extensive Pan2–Pan3 interface links deadenylation to mRNA binding

We identified the linker region between the Pan2 WD40 and UCH domains as a major binding site for Pan3. NanoESI mass spectrometry, isothermal calorimetry, and a crystal structure of the interacting regions of *ct*Pan2 and *ct*Pan3 show that only one Pan2 molecule binds to a Pan3 dimer to form a heterotrimeric complex. This unusual stoichiometry derives from the asymmetry of the Pan3 dimer itself so that the interface to which Pan2 binds is not replicated on the opposite face of Pan3 (Fig5D). The *ct*Pan2 linker wraps around the Pan3 dimer with interactions dominated by the formation of new secondary structural elements (intermolecular β-sheets) and the insertion of Pan2 into surface grooves on Pan3 (Fig6). The extensive interface is consistent with the high enthalpic change observed on binding, and there being a tight interaction (10–50 nM *K*_d_) that is difficult to disrupt.

A previous study using Pan3 mutants defective in dimerization suggested that a single Pan3 chain could bind Pan2 (Christie *et al*, 2013). Although some interaction between the Pan2 PID with a single Pan3 chain may be possible, in our crystal structure, we observed that Pan2 interacts extensively with both chains in the Pan3 dimer. Moreover, conserved solvent-exposed residues in the Pan3 dimer proposed to be involved in Pan2 interactions (Christie *et al*, 2013) do not make contact with the *ct*Pan2 PID in our crystal structure. These regions could participate in additional interactions with Pan2 outside of the PID, in stabilizing the Pan3 structure, or in mediating interactions with other proteins. Consistent with additional Pan2–Pan3 interactions, Pan2 Δ343–406 retained deadenylation activity (Fig7). Furthermore, we found that the conserved N-terminus of the Pan3 pseudokinase, which was truncated in previous studies, changes conformation to form a β-sheet with Pan2.

### Pan2–Pan3 alone shows robust deadenylation activity

Regulation of deadenylation is crucial since it can trigger translational repression and/or rapid mRNA decay. Deadenylation *in vivo* appears to have two phases: an initial phase removes the distal part of the polyA tail until approximately 12–25 As remain (Brown & Sachs, 1998). The proximal polyA tail is then removed during ‘terminal deadenylation’ which triggers mRNA decay (Tucker *et al*, 2001). The recent observation that the median polyA tail length in yeast is approximately 27 nucleotides (Subtelny *et al*, 2014) could imply that at steady state, many mRNAs have undergone distal but not proximal deadenylation. In yeast, deletion of *PAN2* results in long polyA tails (> 50 As), and deletion of *CCR4* impairs removal of the final approximately 20 As (Brown & Sachs, 1998; Tucker *et al*, 2001). Thus, although the complexes appear to be functionally redundant, in the sequential model of deadenylation, Pan2–Pan3 stimulated by PABP performs the initial phase and Ccr4–Not takes over (Brown & Sachs, 1998; Tucker *et al*, 2001; Yamashita *et al*, 2005; Bönisch *et al*, 2007).

In our *in vitro* deadenylation assay, the Pan2–Pan3 complex alone completely deadenylates RNA (Fig1C). PolyA-binding protein stimulates the reaction but is not required, suggesting that this function is intrinsic to the Pan2–Pan3 complex. It is conceivable that Pan2–Pan3 could function independently of Pab1 *in vivo*. Pab1-independent activity of Pan2–Pan3 was observed previously in very low ionic strength conditions and in the presence of spermidine (Lowell *et al*, 1992). Importantly, Pan2–Pan3 retains polyA specificity in the absence of Pab1 (Fig1B and C; Lowell *et al*, 1992). Thus, complete deadenylation can be performed by Pan2–Pan3 alone. This is consistent with suggestions that Pan2–Pan3 and Ccr4–Not may have independent roles (Lowell *et al*, 1992; Decker & Parker, 1993; Fadda *et al*, 2013; Sun *et al*, 2013).

**Figure 8:**
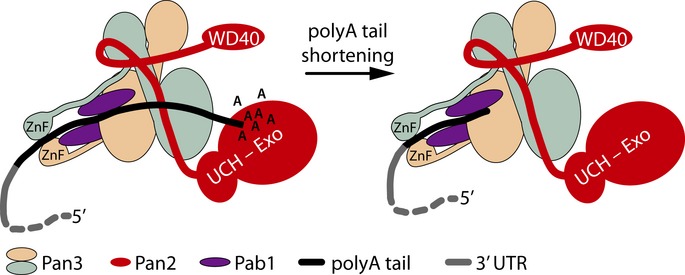
Model for Pan2–Pan3 Schematic model for Pan2–Pan3 function, whereby Pan3 channels RNA to the Pan2 exonuclease domain.

At a molecular level, we propose that the Pan2–Pan3 complex, probably through RNA recognition by Pan3, can distinguish between the polyA tail and the 3′ UTR causing a decrease in the deadenylase activity as the polyA tail shortens. One way in which this could be achieved would be for the distance between the RNA binding regions in the Pan3 dimer to sense when the polyA tail is truncated to approximately < 25 nt. For example, because they are on separate Pan3 chains, it may not be possible for both zinc fingers as well as the PKC to bind and position short polyA stretches for efficient deadenylation by Pan2 (Fig8). In this context, positioning of protein domains within the complex is likely to be important. The formation of a β-sheet between *ct*Pan2 and the N-terminal residues of chain B of the *ct*Pan3 dimer that changes its position dramatically on complex formation could influence the position of the two zinc fingers and the two PAM2 motifs within the complex. The N- and C-termini of the *ct*Pan2 PID are in close proximity in our structure, and this could also have implications for the positioning of the N-terminal WD40 and C-terminal UCH and exonuclease domains of Pan2 on the Pan3 dimer. Other components, including the active site of Pan2, may also contribute to polyA specificity.

In conclusion, through combined biochemical and structural approaches, we have shown that Pan3 is required to couple RNA binding to the Pan2 exonuclease activity. Pan3 binds RNA through multiple mechanisms and forms an extensive interface with Pan2 to ensure a stable interaction for efficient mRNA deadenylation. The zinc fingers, PAM2 motifs, and positively charged surface of Pan3 could channel polyA RNA into the Pan2 exonuclease active site (Fig8). This mechanism of RNA binding could provide Pan2–Pan3 with an intrinsic preference for the polyA tail over the 3′ UTR that would be crucial for its function in the regulation of gene expression.

## Materials and Methods

### Protein expression and purification

Detailed methods of protein expression and purification can be found in Supplementary Methods. In brief, Pan2–Pan3 complexes, Pan2, and Pab1 were expressed in *S. cerevisiae* as described (Galej *et al*, 2013) with a Strep-II affinity tag for purification using StrepTactin affinity chromatography. His-tagged Pan3 zinc finger and GST-Pan3 PKC were expressed in *E. coli* and purified using Ni^2+^ affinity chromatography and glutathione Sepharose, respectively.

### Deadenylation activity assay

A construct containing a T7 promoter, the *CYC1* 3′ UTR (169 nucleotides downstream of the stop codon) followed by 80 adenosines, and a Bsa*I* restriction site were created by gene synthesis and cloned into pUC57 plasmid (GenScript). Following restriction digest with Bsa*I* and mung bean nuclease treatment, the linearized plasmid was used in a run-off *in vitro* transcription reaction. The RNA was eluted from denaturing polyacrylamide gels in 0.5 M ammonium acetate, 1 mM EDTA, 0.2% SDS (w/v) at 37°C overnight and stored in 10 mM ammonium acetate pH 5.4, 50 mM KCl. The *CYC1* 3′ UTR RNA was obtained in the same way from a plasmid lacking the adenosine stretch. Deadenylation reactions were performed in 10 mM HEPES pH 8.0, 50 mM NaCl, 1 mM MgCl_2_, and 5 mM β-mercaptoethanol at 30°C. Reactions contained 5 nM Pan2–Pan3 complex or Pan2, 180 nM (1.7 μg) *CYC1*-A_80_ RNA, and optional 600 nM Pab1 in a total volume of 115 μl and were started by the addition of RNA. 10 μl samples were taken at different time points, and the reaction stopped by addition of 1 mg/ml proteinase K and 25 mM EDTA and incubated at 37°C for 15 min. Reaction products were separated using 6% (w/v) urea polyacrylamide gel electrophoresis and visualized by RNA staining with SYBR Green II (Life Technologies). For Fig1B, the intensity of the RNA down each lane was measured using ImageJ (Schneider *et al*, 2012). For Figs1C and 4C and Supplementary Fig S5C, the average polyA tail length was defined as the maximum intensity in the lane.

### NMR spectroscopy

For the solution NMR structure of *sc*Pan3 zinc finger, complete ^1^H/^13^C/^15^N assignments were obtained and structure determination was carried out using conventional methods. Statistics are summarized in Supplementary Table S1.

### Electrophoretic mobility shift assay

The catalytic activity of *sc*Pan2 was destroyed by mutating Glu912 to Ala using a QuikChange kit (Agilent Technologies). Various concentrations (0.1–3 μM) of inactive *sc*Pan2–Pan3 complex with and without the zinc finger domain were incubated with 40 ng *CYC1* 3′ UTR or *CYC1*-A_80_ in deadenylation assay buffer in a total volume of 10 μl for 15 min at RT. Complex formation was analyzed on 6% NuPage TBE precast gels (Life Technologies), and the RNA stained with SYBR Green II (Life Technologies). The intensity of the free RNA band was measured using ImageJ (Schneider *et al*, 2012).

### Fluorescence polarization

The binding of a 15-mer 5′ Cy3-labeled RNA (polyA, polyC, polyG and polyU), at a final concentration of 20 nM, to a dilution series of *ct*Pan3 PKC in 10 mM Tris pH 8.0, 50 mM NaCl, 1 mM MgCl_2_, 5 mM β-mercaptoethanol was measured, in triplicate, by fluorescence polarization in a PHERAstar Plus instrument (BMG Labtech). Purified GST was used as a negative control. The background (protein without RNA) was subtracted.

To compare RNA binding of *ct*Pan2, *ct*Pan3 PKC, and the *ct*Pan2–Pan3 PKC complex, a catalytically inactive *ct*Pan2 mutant was made by QuikChange (Agilent Technologies) mutating Glu 899 to Ala. The assay described above was performed using a 25-mer of polyA RNA.

### Crystallization and structure determination methods

Crystals of *ct*Pan3 and *ct*Pan2–Pan3 were obtained by vapor diffusion, and data were collected, processed, and refined as described in Supplementary Methods. The refinement statistics are summarized in Supplementary Tables S2 and S3.

### Analytical size exclusion

Separately purified *ct*Pan2 constructs containing deletions of parts of the PID domain and *ct*Pan3 PKC dimer were mixed in a 1:1 ratio at 1 μM concentration and incubated at room temperature for 15 min. Fifty microliter samples were injected into a Superdex 200 Increase 3.2/300 size exclusion column (GE Healthcare) pre-equilibrated in 20 mM Tris pH 8.0, 500 mM NaCl, 2 mM TCEP. 65 μl fractions were collected in all experiments and analyzed on 4–12% NuPage Bis–Tris precast gels (Life Technologies). Gels were stained with Coomassie brilliant blue, and protein band intensities measured using ImageJ (Schneider *et al*, 2012).

### Nanoelectrospray ionization (nanoESI) mass spectrometry (MS)

NanoESI mass spectrometry experiments were performed on a high mass Q-TOF-type instrument (Chernushevich & Thomson, 2004) adapted for a QSTAR XL platform (MDS Sciex) (Chernushevich & Thomson, 2004). Prior to MS analysis, 50 μl of 5.5 mg/ml *sc*Pan2–Pan3 complex was buffer-exchanged into 200 mM ammonium acetate solution, pH 8.0 twice using Bio-Rad Micro Bio-Spin columns (P-6). Protein solution was loaded for sampling via gold-plated borosilicate glass capillaries made in-house as described previously (Nettleton *et al*, 1998). The following experimental parameters were used: capillary voltage 1.3 kV, declustering potential 100 V, focusing potential 150 V, second declustering potential 15 V, and focusing rod offset varied from 80 to 100 V, MCP 2,550. Argon was used as a collision gas at maximum pressure. All spectra were calibrated externally using a solution of cesium iodide (100 mg/ml).

### Accession codes

Sequences of two Pan3 isoforms cloned from a *Chaetomium thermophilum* cDNA library have been deposited in GenBank with Accession codes KJ657770 (full-length Pan3) and KJ657771 (splice variant without N-terminal zinc finger). For the three structures reported, the final coordinates have been deposited in the Protein Data Bank with PDB accession codes 4CYI (*ct*Pan3 PKC), 4CYJ (*ct*Pan3 PKC–*ct*Pan2 PID), and 4CYK (*sc*Pan3 zinc finger).
