# Supplementary material for: Structural basis for Pan3 binding to Pan2 and its function in mRNA recruitment and deadenylation
Source: EMBO J. 2014 May 29;33(14):1514–26. doi: 10.15252/embj.201488373 (PMC4158885; doi:10.15252/embj.201488373)
Supplement: Supplementary file 1 [file embj0033-1514-sd1.pdf]

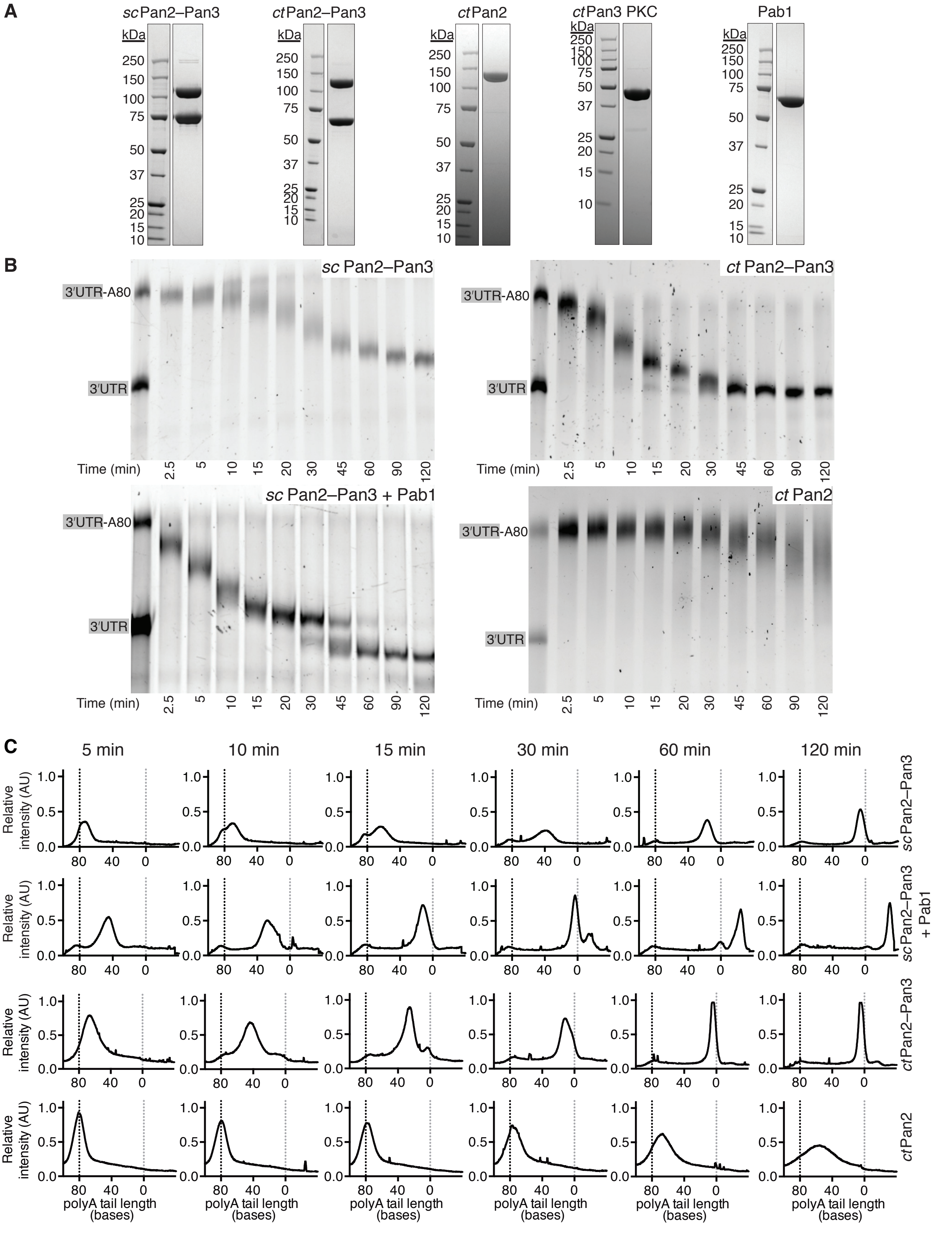

Supplementary Figure S1: Deadenylation activity of the Pan2–Pan3 complex does not require PABP/Pab1

A Coomassie-blue stained SDS-polyacrylamide gels of recombinant purified proteins.

B-C Deadenylation assays of an RNA substrate (*CYC1* 3′ UTR with A_{80} tail) by recombinant Pan2–Pan3 from *S. cerevisiae* (*sc*) in the presence and absence of Pab1, Pan2–Pan3 from *C. thermophilum* (*ct*) and isolated *ct*Pan2. Time points were analyzed by denaturing polyacrylamide gel electrophoresis, shown in (**B**). The first lane shows *CYC1* 3′ UTR with and without A_{80} tail as a marker. Lane intensities of selected time points are plotted separately in panel (**C**).
