# Supplementary material for: Structural basis for Pan3 binding to Pan2 and its function in mRNA recruitment and deadenylation
Source: EMBO J. 2014 May 29;33(14):1514–26. doi: 10.15252/embj.201488373 (PMC4158885; doi:10.15252/embj.201488373)
Supplement: Supplementary file 2 [file embj0033-1514-sd2.pdf]

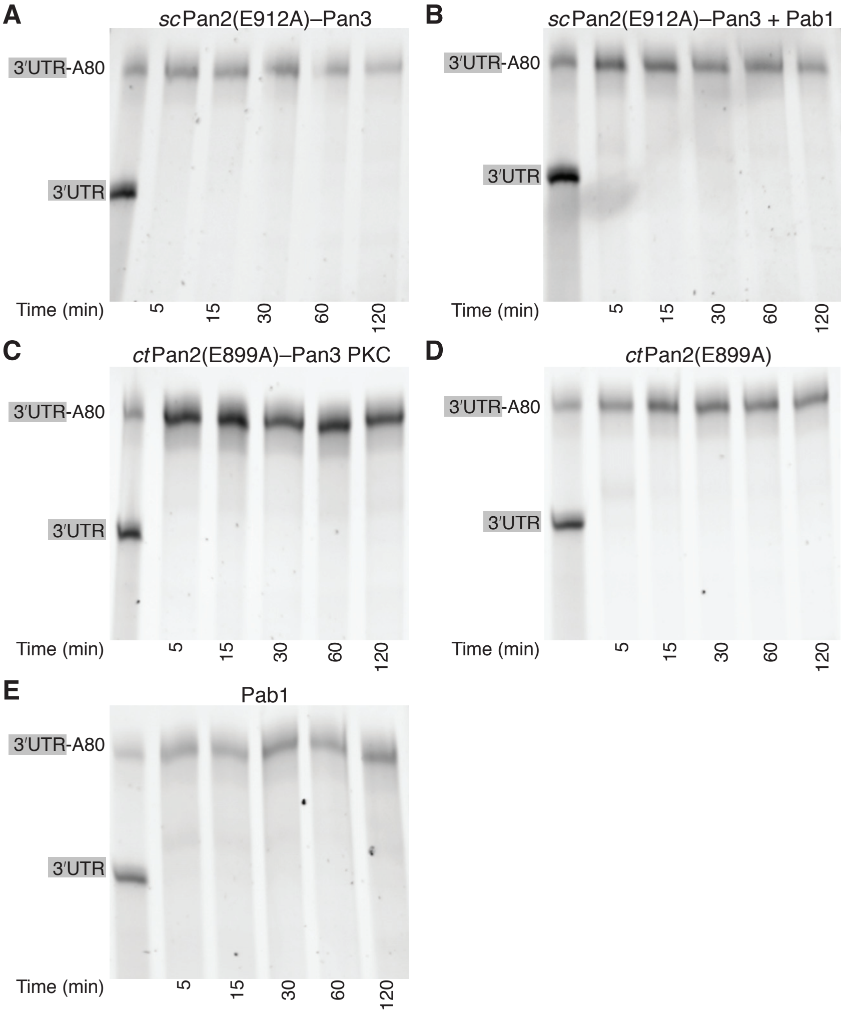

Supplementary Figure S2: Active site mutants of the Pan2–Pan3 complex are not active in deadenylation assays.

Deadenylation assays of an RNA substrate (*CYC1* 3′ UTR with A_{80} tail) by recombinant Pan2–Pan3 from *S. cerevisiae* (*sc*) with an active site mutation in Pan2 (E912A) in the absence (**A**) and presence (**B**) of Pab1, Pan2–Pan3 from *C. thermophilum* (*ct*) with an active site mutation in Pan2 (E899A) (**C**), isolated *ct*Pan2-E899A (**D**) and isolated *sc*Pab1 (**E**). Time points were analyzed by denaturing polyacrylamide gel electrophoresis. The first lane of each gel shows *CYC1* 3′ UTR with and without A_{80} tail as a marker.
