# Supplementary material for: Structural basis for Pan3 binding to Pan2 and its function in mRNA recruitment and deadenylation
Source: EMBO J. 2014 May 29;33(14):1514–26. doi: 10.15252/embj.201488373 (PMC4158885; doi:10.15252/embj.201488373)
Supplement: Supplementary file 3 [file embj0033-1514-sd3.pdf]

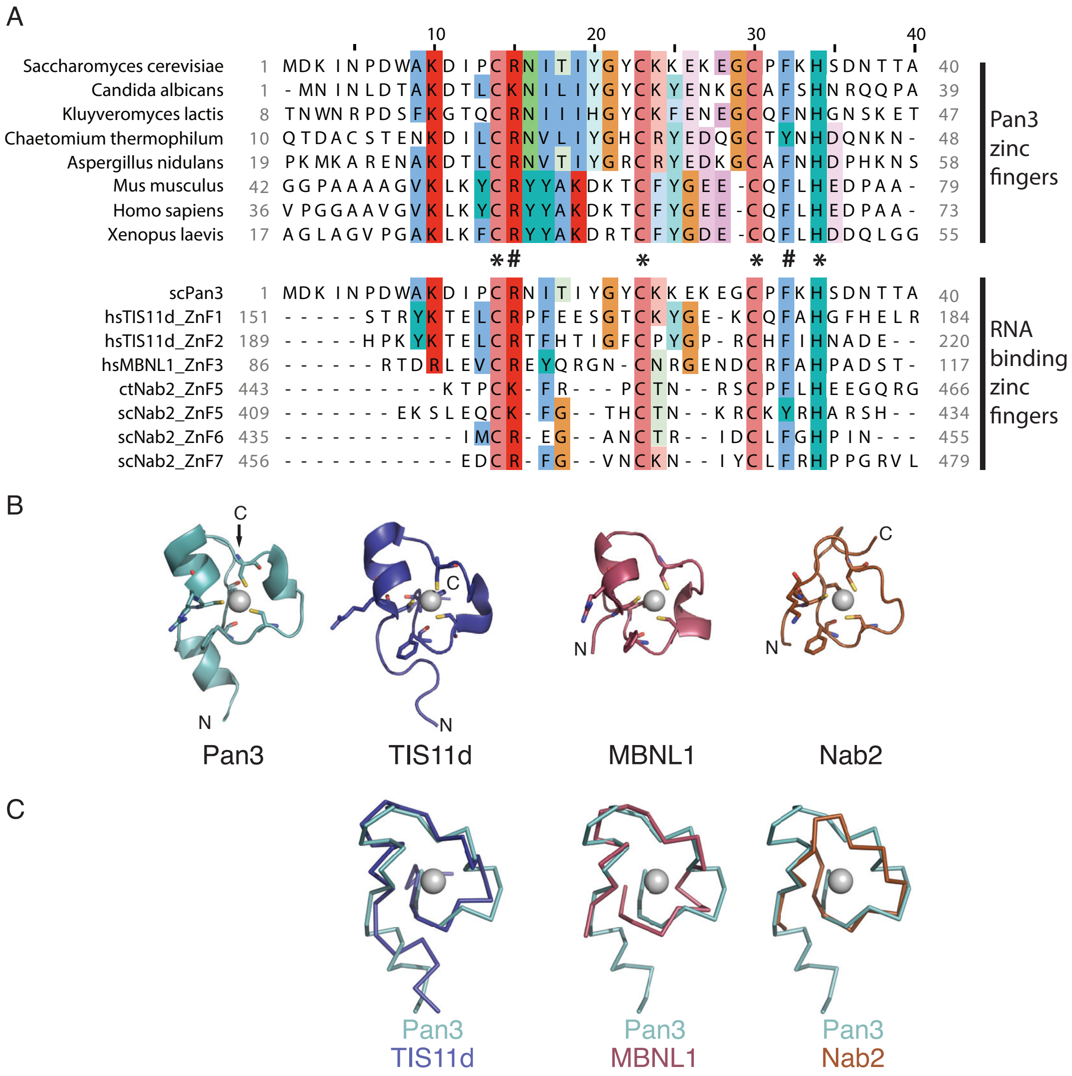

Supplementary Figure S3: Comparison of the *sc*Pan3 zinc finger to other zinc fingers.

A Sequence alignment of Pan3 zinc fingers from different species (top) and with other RNA-binding zinc fingers (bottom). Zinc co-ordinating residues are indicated with asterisks and other highly conserved residues are marked with hashes. sc, *Saccharomyces cerevisiae*; hs, *Homo sapiens*; ct, *Chaetomium thermophilum*.

B Structures of the zinc finger of *sc*Pan3 (this study), zinc finger 2 of human TIS11d (PDB 1rgo, Hudson *et al*, 2004), zinc finger 3 of human MBNL1 (PDB 3d2s, Teplova & Patel, 2008) and zinc finger 5 of *C. thermophilum* Nab2 (PDB 4lj0, Kuhlmann *et al*, 2014) in cartoon representation. The zinc coordinating residues (Cys–Cys–Cys–His) and conserved Arg/Lys and Phe are shown in stick representation. N- and C-termini are indicated.

C Superposition of *sc*Pan3 zinc finger with TIS11d, MBNL1 and Nab2 (in ribbon representation).

Structures of TIS11d, MBNL1 and Nab2 bound to RNA provide a model for RNA binding (Hudson *et al*, 2004; Teplova & Patel, 2008; Kuhlmann *et al*, 2014). Each of these fingers binds one to four RNA bases and in all cases, the bases are inserted into surface grooves on the molecule where they stack against aromatic or basic side chains and hydrogen bond to the protein main chain or zinc finger cysteines. A conserved Arg or Lys and a hydrophobic amino acid (Phe or Tyr) are often involved in stacking interactions with RNA bases. The equivalent residues are conserved in Pan3 zinc fingers (Arg15 and Phe32) and experience chemical shifts upon incubation with polyA RNA (Fig 2C and S4). An upstream "lead-in" sequence in TIS11d is also important for RNA binding providing positive charge to neutralize the RNA backbone and a conserved tyrosine which undergoes base stacking with the RNA (Hudson *et al*, 2004; Lai *et al*, 2014). In Pan3, the large chemical shift in Trp8, Arg15 and their surrounding residues after polyA addition is consistent with a similar mechanism. A structure of *C. thermophilum* Nab2 zinc fingers bound to polyA RNA explains its specificity for adenosine with hydrogen bonds between purine N6 and one of the zinc-coordinating cysteines, and between N7 or N1 and the main chain atoms of the protein (Kuhlmann *et al*, 2014). Guanine does not bind efficiently because the O6 atom cannot hydrogen bond to cysteine. By analogy, there may be a hydrogen bond between an adenine N6 and the thiol of Pan3 Cys23 that displays a change in chemical shift on binding polyA (Fig 2C).
