# Supplementary material for: Structural basis for Pan3 binding to Pan2 and its function in mRNA recruitment and deadenylation
Source: EMBO J. 2014 May 29;33(14):1514–26. doi: 10.15252/embj.201488373 (PMC4158885; doi:10.15252/embj.201488373)
Supplement: Supplementary file 4 [file embj0033-1514-sd4.pdf]

A

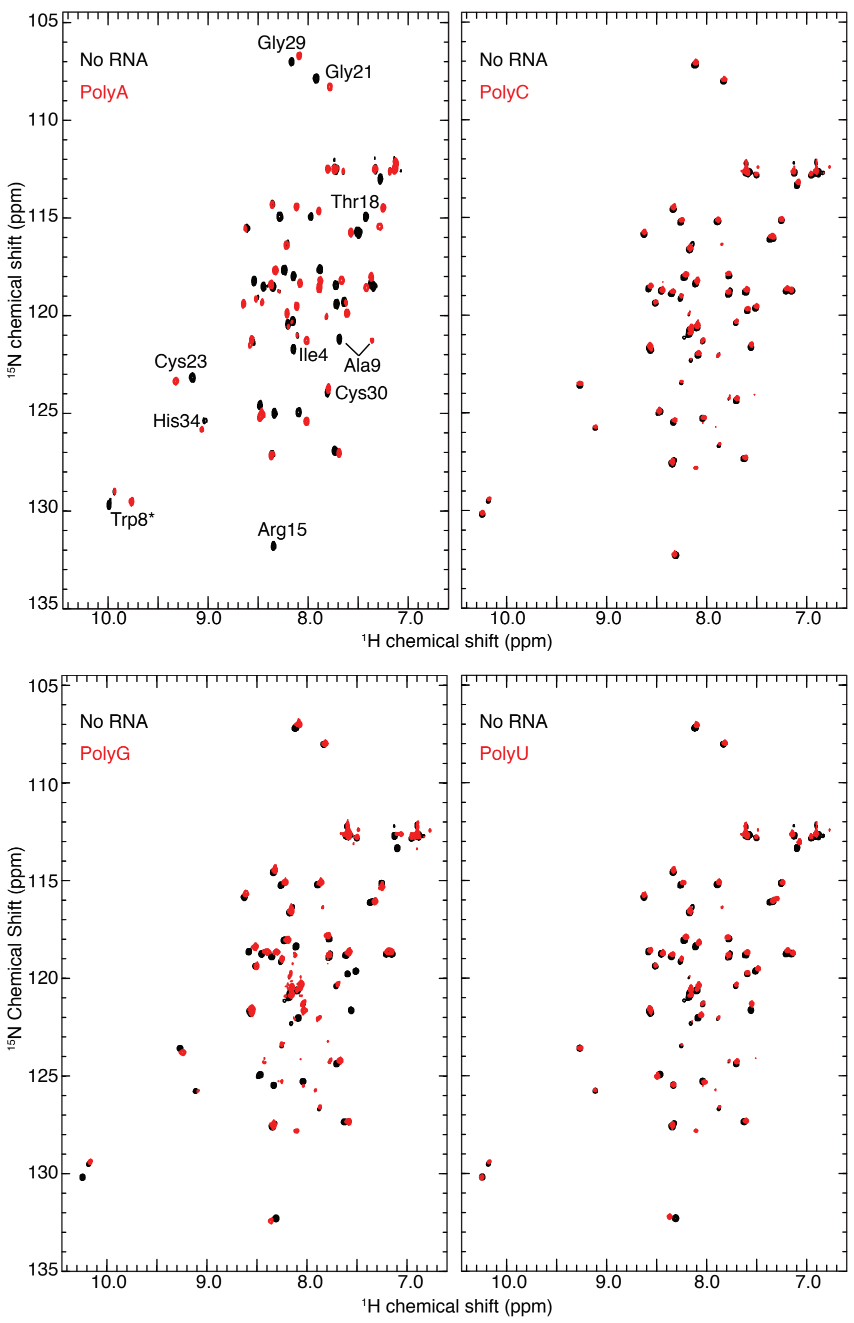

B

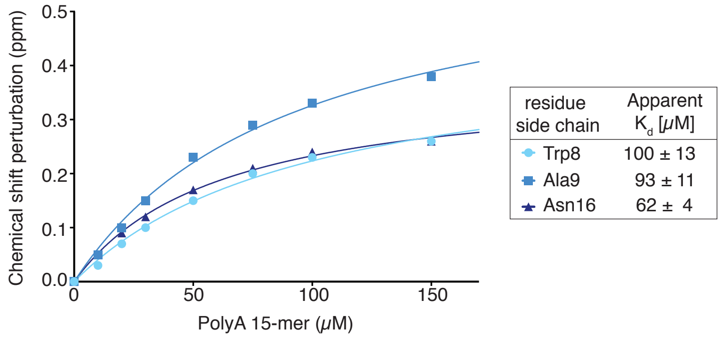

Supplementary Figure S4: The zinc finger of *sc*Pan3 binds polyA RNA.

A ^{15}N-^{1}H HSQC spectra of 150 μM *sc*Pan3 zinc finger (black), and the same sample containing 150 μM 15-mer RNA (red). Selected residues are labeled. Trp8 has two conformations (marked by an asterisk).

B Titration of 15-mer polyA RNA to *sc*Pan3 zinc finger. Chemical shift perturbations of Trp8, Ala9 and Asn16 are plotted against RNA concentration. The solid line represents the fit of the data. Apparent K_d for each residue is indicated (± standard error).
