# Supplementary material for: Structural basis for Pan3 binding to Pan2 and its function in mRNA recruitment and deadenylation
Source: EMBO J. 2014 May 29;33(14):1514–26. doi: 10.15252/embj.201488373 (PMC4158885; doi:10.15252/embj.201488373)
Supplement: Supplementary file 5 [file embj0033-1514-sd5.pdf]

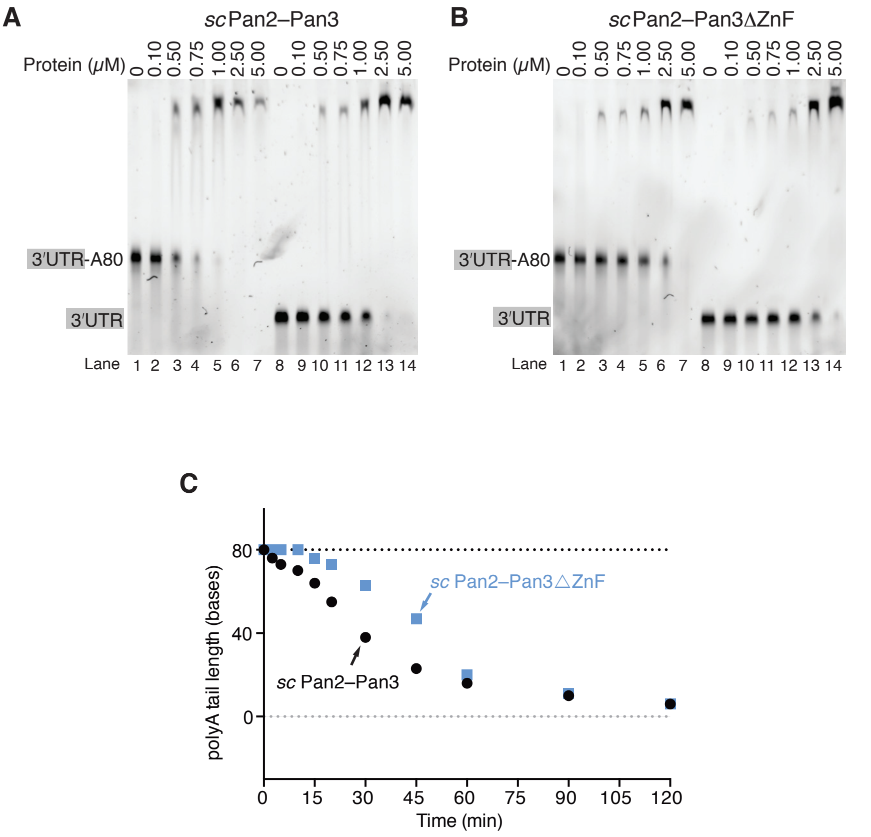

Supplementary Figure S5: The zinc finger domain of *sc*Pan3 increases the affinity of the Pan2–Pan3 complex to polyA RNA.

A-B Electrophoretic mobility shift assay (EMSA) using the *CYC1* 3′ UTR RNA with (lanes 1-7) or without (lanes 8-14) a polyA$_{80}$ tail. RNA was incubated with (**A**) *sc*Pan2–Pan3 or (**B**) *sc*Pan2–Pan3 with a deletion of the Pan3 zinc finger (*sc*Pan2–Pan3ΔZnF). Both complexes contain an active site mutation in *sc*Pan2 (E912A). Binding was analyzed by native polyacrylamide gel electrophoresis.

C Deletion of the zinc finger domain results in impaired polyA tail removal. Deadenylation assays using the *CYC1* 3′ UTR with polyA$_{80}$ tail as substrate with *sc*Pan2–Pan3 (black) and *sc*Pan2–Pan3 with a deletion of the zinc finger (blue). Average polyA tail length is plotted for each time point. Dotted lines indicate the sizes of the *CYC1* 3′ UTR RNA with (black) and without (grey) a polyA$_{80}$ tail.
