# Supplementary material for: Structural basis for Pan3 binding to Pan2 and its function in mRNA recruitment and deadenylation
Source: EMBO J. 2014 May 29;33(14):1514–26. doi: 10.15252/embj.201488373 (PMC4158885; doi:10.15252/embj.201488373)
Supplement: Supplementary file 6 [file embj0033-1514-sd6.pdf]

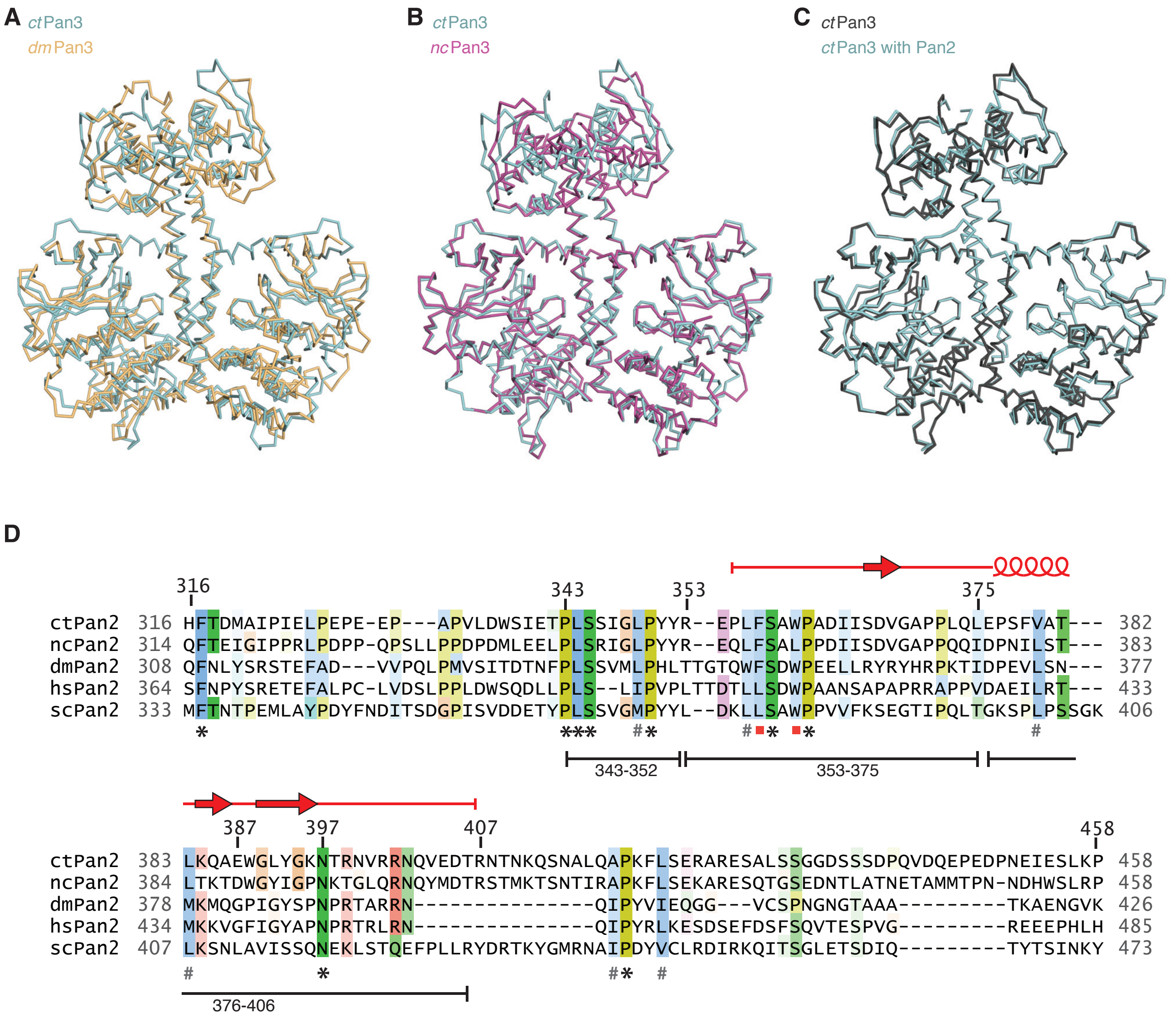

Supplementary Figure S6: Structural and sequence comparison of Pan3 proteins from different species.

A-C Structures of Pan3 were overlaid and are displayed in ribbon representation for (**A**) *Chaetomium thermophilum* (*ct*, cyan; this study) and *Drosophila melanogaster* (*dm*, gold; Christie *et al*, 2013) Pan3; (**B**) *ct* (cyan) and *Neurospora crassa* (*nc*, pink; Christie *et al*, 2013) Pan3; and (**C**) isolated *ct*Pan3 (grey) and *ct*Pan3 bound to *ct*Pan2 (cyan, *ct*Pan2 not shown; both this study).

D Sequence alignments of Pan2 PID (Pan3-interacting domain). Highly conserved residues are indicated with asterisks, conserved hydrophobic residues F357 and W360 are marked with red squares, and other conserved residues are marked with hashes. Residues observed in the crystal structure are indicated by a red line and secondary structure elements are shown as arrows and spiral (β-strands and α-helices). Residue numbers used for truncation constructs in Supplementary Fig 7D-E are shown and for deletions in Fig. 7 are indicated by black bars under the alignment. *ct*, *Chaetomium thermophilum*; *nc*, *Neurospora crassa*; *dm*, *Drosophila melanogaster*; *hs*, *Homo sapiens*; *sc*, *Saccharomyces cerevisiae*.
