# Supplementary material for: Structural basis for Pan3 binding to Pan2 and its function in mRNA recruitment and deadenylation
Source: EMBO J. 2014 May 29;33(14):1514–26. doi: 10.15252/embj.201488373 (PMC4158885; doi:10.15252/embj.201488373)
Supplement: Supplementary file 7 [file embj0033-1514-sd7.pdf]

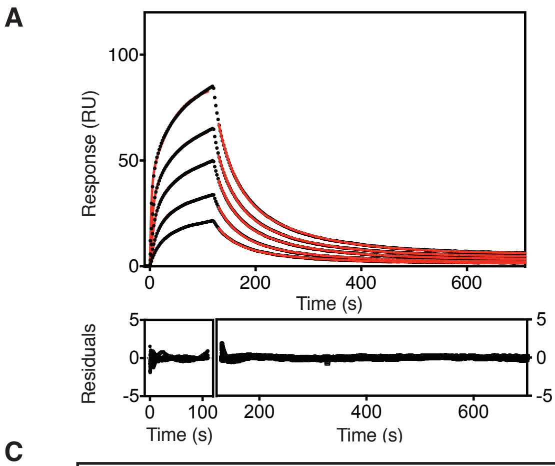

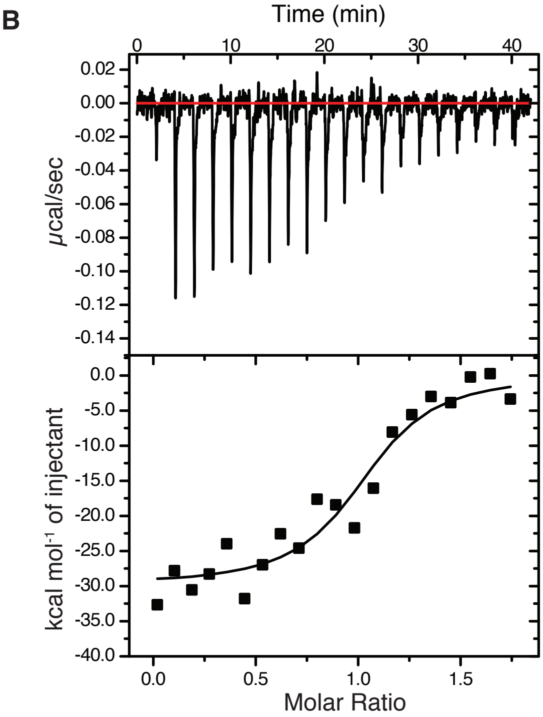

C

SPR	K_d (nM)	k_a (M^{-1} s^{-1})	k_d (s^{-1})
ct Pan2$^{343\text{-}458}$	10 ± 0.1	$3.1\times10^6 \pm 0.02\times10^6$	0.031 ± 0.002

ITC	K_d (nM)	△H (kcal mol^{-1})	-T△S (kcal mol^{-1})
ct Pan2$^{343\text{-}458}$	49 ± 21	-29.8 ± 1.5	19.9

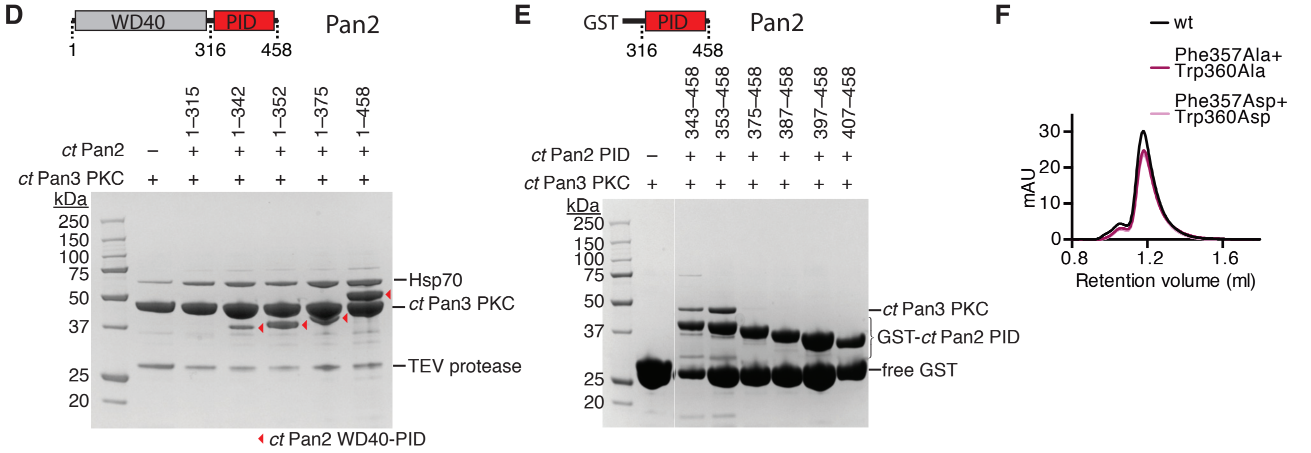

Supplementary Figure S7: *ct*Pan2 PID binds to *ct*Pan3.

A Surface plasmon resonance (SPR) experiment measuring the affinity of the interaction between immobilized GST-*ct*Pan2$^{343\text{-}458}$ and *ct*Pan3 PKC dimer. GST-*ct*Pan2 was bound to the chip using an anti-GST antibody and incubated with 5 different concentrations of *ct*Pan3 PKC dimer. The experiment was performed in triplicate. A representative graph of the fitted data is shown.

B Isothermal titration calorimetry (ITC). The excess heat measured during the titration of *ct*Pan3 PKC dimer into GST-*ct*Pan2$^{343\text{-}458}$ at 25 °C (upper panel) was integrated and plotted against the molar ratios of the two binding partners (lower panel). The solid line represents the fit of the data to a single-site binding model.

C Summary of binding data obtained from SPR and ITC experiments. Standard errors are given.

D Pull-downs of GST-tagged *ct*Pan3 PKC co-expressed in *Escherichia coli* with *ct*Pan2 constructs containing the WD40 and PID regions with sequential C-terminal truncations. Proteins were eluted by cleaving the GST-tag with TEV protease, and then analyzed by SDS-PAGE and Coomassie-blue staining. Bound *ct*Pan2 WD40-PID is indicated by red arrowheads. The binding analysis indicates that C-terminal truncations into the PID domain weaken Pan2–Pan3 interaction only after removal of all but 27 residues. Removal of the entire linker (1-315) is necessary to prevent the interaction completely. Contaminating Hsp70 and TEV protease are indicated.

E GST-tagged *ct*Pan2 PID domain constructs containing N-terminal truncations were bound to Glutathione Sepharose then incubated with purified *ct*Pan3 PKC and washed extensively. Bound fractions were analyzed by SDS-PAGE. These pull-downs indicate that removal of at least the first 59 residues of the PID (375-458) is necessary to impair the interaction between Pan2 and Pan3. Even though the lanes are overloaded with GST-*ct*Pan2 PID, binding to *ct*Pan3 PKC is not detected for constructs lacking these residues

F Mutation of highly-conserved aromatic residues Phe357 and Trp360 that bind pockets in the *ct*Pan3 PKC dimer (see Fig. 6C) does not disrupt the Pan2–Pan3 interaction. Separately purified *ct*Pan2 point mutants and *ct*Pan3 PKC were mixed and binding was analyzed by size exclusion chromatography. Elution profiles measuring $A_{280\ nm}$ are compared to wild type (wt) *ct*Pan2–Pan3 PKC.
