# Supplementary material for: Structural basis for Pan3 binding to Pan2 and its function in mRNA recruitment and deadenylation
Source: EMBO J. 2014 May 29;33(14):1514–26. doi: 10.15252/embj.201488373 (PMC4158885; doi:10.15252/embj.201488373)
Supplement: Supplementary file 8 [file embj0033-1514-sd8.pdf]

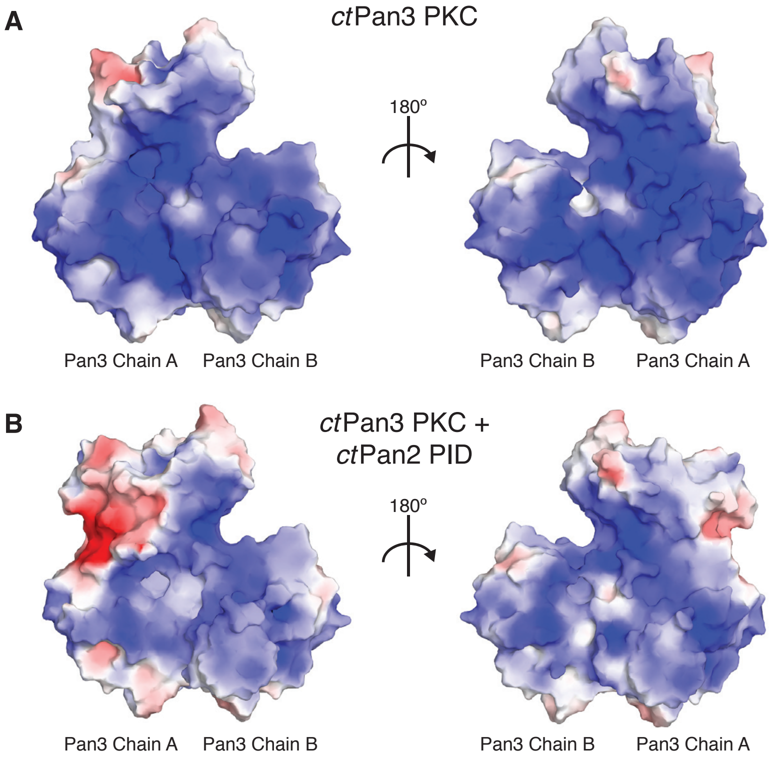


**Supplementary Figure S8: Electrostatic surface potential of the *ct*Pan2 PID-*ct*Pan3 PKC complex.**

The solvent-accessible surface of complex is coloured according to the electrostatic potential, ranging from deep blue (positive charge) to red (negative charge) in the range ± 5 kT/e. The electrostatic potentials were calculated by solving the Poisson–Boltzmann equation with the APBS plugin in PyMOL using the default settings.
