# Supplementary material for: Structural basis for Pan3 binding to Pan2 and its function in mRNA recruitment and deadenylation
Source: EMBO J. 2014 May 29;33(14):1514–26. doi: 10.15252/embj.201488373 (PMC4158885; doi:10.15252/embj.201488373)
Supplement: Supplementary file 9 [file embj0033-1514-sd9.pdf]

Supplementary Table S1: Summary of conformational constraints and statistics for the 20 accepted NMR structures of the Pan3 zinc finger domain

**Structural constraints**	
Intra-residue	317
Sequential	239
Medium-range (2 ≤ | i–j | ≤ 4)	230
Long-range (| i–j | > 4)	149
Dihedral angle constraints	8
TALOS constraints	62
Distance constraints for 4 hydrogen bonds	8
Zinc co-ordination constraints	10
Total	1023

**Statistics for accepted structures**	
Statistics parameter (±SD)	
RMS deviation for distance constraints	0.0082Å ± 0.0005Å
RMS deviation for dihedral constraints	0.390° ± 0.021°
Mean CNS energy term (kcal mol^{-1} ±SD)	
E (overall)	69.29 ± 2.12
E (van der Waals)	29.49 ± 1.34
E (distance constraints)	4.81 ± 0.53
E (dihedral and TALOS constraints)	1.30 ± 0.14
RMS deviations from the ideal geometry (±SD)	
Bond lengths	0.0019 Å ± 0.0005 Å
Bond angles	0.392° ± 0.0028°
Improper angles	0.250° ± 0.011°
Average atomic RMSD from the mean structure (±SD)	
Residues 4-35 (N, Cα, C atoms)	0.193 Å ± 0.061 Å
Residues 4-35 (all heavy atoms)	0.780 Å ± 0.076 Å
