# Supplementary material for: Structural basis for Pan3 binding to Pan2 and its function in mRNA recruitment and deadenylation
Source: EMBO J. 2014 May 29;33(14):1514–26. doi: 10.15252/embj.201488373 (PMC4158885; doi:10.15252/embj.201488373)
Supplement: Supplementary file 10 [file embj0033-1514-sd10.pdf]

Supplementary Table S2: Pan3 crystallographic data collection and refinement statistics.

**Data collection statistics**	
Wavelength (Å)	0.9795
Space group	*P*1
Unit cell parameters: a, b, c (Å); α, β, γ (°)	51.5, 146.0, 149.9; 89.8, 81.1, 82.8
Resolution range (outer shell in brackets; Å)	52.21 – 2.42 (2.48 – 2.42)
Unique reflections	160,956
Total observations	1,170,731
<I / σ(I)>: all (outer shell)	14.3 (2.4)
$R_{p.i.m.}$: all (outer shell)	4.0 (35.9)
Completeness: all (outer shell) (%)	98.5 (98.0)
Multiplicity	7.3
Wilson B-factor	49.1

**Refinement statistics**	
Non-hydrogen atoms	27,217
Metal ions	8 × Mg^{2+}
Ligands	8 × ATP
Number of water molecules	984
Bond length deviation from ideal values (Å)	0.004
Bond angle deviation from ideal values (°)	0.875
Ramachandran favoured/outliers (%)	99.1/0.0
All-atom clashscore	3.28
Reflections used in refinement	160,878
Random reflections assigned for cross-validation	8,073
R_{work}/R_{free} (%)	19.0/23.4
Estimate of the coordinate error (Å)	0.3
