# Supplementary material for: Structural basis for Pan3 binding to Pan2 and its function in mRNA recruitment and deadenylation
Source: EMBO J. 2014 May 29;33(14):1514–26. doi: 10.15252/embj.201488373 (PMC4158885; doi:10.15252/embj.201488373)
Supplement: Supplementary file 11 [file embj0033-1514-sd11.pdf]

Supplementary Table S3: Pan3:Pan2 crystallographic data collection and refinement statistics.

**Data collection statistics**	
Wavelength (Å)	0.9763
Space group	$P2_1$
Unit cell parameters: a, b, c (Å); α, β, γ (°)	89.0, 145.3, 101.8; 90.0, 94.8, 90.0
Resolution range (outer shell in brackets; Å)	100.0 – 2.59 (2.68 – 2.59)
Unique reflections	80,131
Total observations	525,003
$\langle I / \sigma(I) \rangle$: all (outer shell)	12.0 (1.77)
$R_{p.i.m.}$: all (outer shell)	6.0 (66.0)
Completeness: all (outer shell) (%)	99.8 (99.7)
Multiplicity	6.5
Wilson B-factor	62.5

**Refinement statistics**	
Non-hydrogen atoms	14,282
Metal ions	4 × Mg^{2+}
Ligands	4 × ATP
Number of water molecules	150
Bond length deviation from ideal values (Å)	0.006
Bond angle deviation from ideal values (°)	1.146
Ramachandran favoured/outliers (%)	99.4/0.0
All-atom clashscore	2.62
Reflections used in refinement	80,119
Random reflections assigned for cross-validation	4,021
R_{work}/R_{free} (%)	18.3/22.1
Estimate of the coordinate error (Å)	0.3
