# Supplementary material for: Structural basis for Pan3 binding to Pan2 and its function in mRNA recruitment and deadenylation
Source: EMBO J. 2014 May 29;33(14):1514–26. doi: 10.15252/embj.201488373 (PMC4158885; doi:10.15252/embj.201488373)
Supplement: Supplementary file 12 [file embj0033-1514-sd12.pdf]

SUPPLEMENTARY METHODS

Protein expression and purification

Genes encoding proteins from *Saccharomyces cerevisiae (sc)* were cloned from genomic DNA; the *Chaetomium thermophilum (ct)* Pan3 gene was amplified from cDNA; and the gene encoding *ct*Pan2 was synthesized. Expression in *S. cerevisiae* was as described (Galej *et al*, 2013). The following proteins were cloned into pUC18 vectors containing an expression cassette (Wagenbach *et al*, 1991): *sc*Pan2, *sc*Pan3 with C-terminal StrepII-tag, *sc*Pan3 Δzinc finger (42-679) with C-terminal StrepII-tag, *sc*Pab1 with N-terminal StrepII-tag, *ct*Pan2 with N-terminal StrepII-His-tag, *ct*Pan3 and *ct*Pan3 PKC (205-640). The expression cassettes were transferred to pRS426 (*sc*Pan2 and *ct*Pan2) and pRS424 (*sc*Pan3, *sc*Pan3 Δzinc finger, *ct*Pan3, *ct*Pan3 PKC and *sc*Pab1) plasmids (Christianson *et al*, 1992). *Saccharomyces cerevisiae* BCY123 host cells (*MATa pep4::HIS3 prb1::LEU2 bar1::HIS6 lys2::GAL1/10GAL4 can1 ade2 trp1 ura3 his3 leu23,112)* transformed with both plasmids were grown in –URA –TRP selective medium to an OD_{600nm} = 0.8–1.2. Protein expression was induced with 2% (w/v) galactose and cells grown for 12–16 hours at 30 °C.

All protein purifications were carried out at 4 °C. Cells expressing *sc*Pan2–Pan3 complexes were resuspended in 50 mM PIPES pH 6.5, 300 mM NaCl, 5 mM β-mercaptoethanol, EDTA-free protease inhibitor tablets (Roche) and DNaseI and lysed using a cell disruptor (Constant Systems Ltd) at 35 kPsi. Cell lysates

were cleared by centrifugation and bound to StrepTactin resin (IBA). Proteins were eluted in lysis buffer containing 5 mM desthiobiotin. *sc*Pan2–Pan3 complexes were diluted 1:5 using 20 mM PIPES pH 6.5 and 5 mM β-mercaptoethanol and subjected to cation exchange (ResourceS; GE Healthcare) and size exclusion chromatography (HiLoad Superdex 200 26/60; GE Healthcare). Proteins were stored in 20 mM CHES pH 9.0, 150 mM NaCl, 5 mM β-mercaptoethanol.

*ct*Pan2 constructs and *ct*Pan2–Pan3 complexes were lysed in 50 mM Tris pH 8.0, 500 mM NaCl, 2 mM TCEP, EDTA-free protease inhibitor tablets (Roche) and DNaseI and purified using StrepTactin affinity chromatography as described above. Further purification was achieved using Ni^{2+}-affinity and size exclusion chromatography (HisTRAP HP and HiLoad Superdex 200 16/60; GE Healthcare). Proteins were stored in 20 mM Tris pH 8.0, 500 mM NaCl, 2 mM TCEP.

Pab1 was lysed in 50 mM HEPES pH 8.0, 300 mM NaCl, 5 mM β-mercaptoethanol, EDTA-free protease inhibitor tablets (Roche) and DNaseI and purified using StrepTactin affinity chromatography as described above. Protein in the elution peak was bound to hydrophobic interaction column (HiTRAP Phenyl HP; GE Healthcare) in 1 M $(NH_4)_2SO_4$ and eluted in a gradient to 20 mM HEPES pH 7.5, 150 mM NaCl and 2 mM β-mercaptoethanol. As a final step, size

exclusion chromatography (HiLoad Superdex 200, GE Healthcare) in 20 mM HEPES pH 7.5, 150 mM NaCl and 2 mM β-mercaptoethanol was performed.

A *ct*Pan3 construct encoding the pseudokinase and C-terminal domains (Pan3 PKC, residues 205-640) were cloned into a pGEX-TEV vector. For expression of *ct*Pan2 PID–Pan3 PKC complex, GST-*ct*Pan3 PKC was subcloned into pET-Duet-1 (Novagene) together with various *ct*Pan2 PID constructs. Proteins were expressed in *Escherichia coli* BL21 (DE3) cells grown in 2xTY medium at 37 °C to OD_{600nm} = 0.6. The expression was induced with 1 mM IPTG and cells were grown for another 3 hours at 23 °C. Bacterial cells were lysed by sonication in 50 mM Tris pH 7.4, 500 mM NaCl, 2 mM TCEP, EDTA-free protease inhibitor tablets (Roche) and DNaseI. Lysates were cleared by centrifugation and incubated with glutathione Sepharose (GE Healthcare) for 1 hour. Bound proteins were eluted in 20 mM Tris pH 8.0, 500 mM NaCl, 20 mM glutathione and 2 mM TCEP and the GST-tag was cleaved using TEV protease at 4 °C overnight. Contaminating TEV and GST were removed on a HiLoad Superdex 200 26/60 column (GE Healthcare) in 20 mM Tris pH 7.4, 500 mM NaCl and 2 mM TCEP. Selenomethionine-derivatized *ct*Pan3 PKC was expressed in *Escherichia coli* C41 cells grown in a modified K-MOPS minimal media (Neidhardt *et al*, 1974) at 37 °C to OD_{600nm} = 0.6. Amino acids lysine, threonine, phenylalanine, leucine, isoleucine, valine and selenomethionine were added 30 min before induction with 1 mM IPTG. Cells were grown for another 5 hours at

37 °C. Selenomethionine-derivatized protein was purified as described above except 5 mM TCEP was used in all buffers. All proteins were snap frozen in liquid nitrogen and stored at -80 °C.

For NMR spectroscopy, the DNA sequence encoding the *sc*Pan3 zinc finger domain (residues 1-41) was cloned into a modified pRSETa (Invitrogen) expression vector (Dodd *et al*, 2004). The protein was expressed in *Escherichia coli* C41 host cells and grown in 2xTY medium. The cultures were induced with 1 mM IPTG at an OD_{600nm} = 0.8 and harvested after 16 hours at 22 °C by centrifugation. Isotopically-labelled Pan3 zinc finger was prepared by growing cells in a modified K-MOPS minimal media (Neidhardt *et al*, 1974) containing $^{15}NH_4Cl$ and/or [^{13}C]-glucose. The resulting protein was purified by Ni^{2+}-NTA affinity chromatography, TEV protease digestion and dialysis and a second Ni^{2+}-NTA affinity chromatography to remove the lipoyl domain fusion tag. Final purification was performed with size exclusion chromatography using a HiLoad 26/60 Superdex 30 column (GE Healthcare).

The theoretical pI was calculated using ProtParam on the ExPASy server (http://web.expasy.org/protparam/). Sequence alignments were performed with Clustal Omega (Sievers *et al*, 2011) and figures were prepared with JalView (Waterhouse *et al*, 2009).

NMR spectroscopy

Protein samples prepared for NMR spectroscopy experiments were typically 1.5 mM in 90% (v/v) H_2O, 10% (v/v) D_2O, containing 20 mM Tris pH 7.0, 100 mM NaCl and 5 mM d-β-mercaptonethanol. All spectra were acquired using either a Bruker DRX800 or DRX600 spectrometers equipped with pulsed field gradient triple resonance at 25 °C, and referenced relative to external sodium 2,2-dimethyl-2-silapentane-5-sulfonate (DSS) for proton and carbon signals, or liquid ammonium for that of nitrogen. Assignments were obtained using standard NMR methods using $^{13}C/^{15}N$-labelled, ^{15}N-labelled, 10% ^{13}C-labelled and unlabelled Pan3 zinc finger NMR samples (Bax *et al*, 1991; Englander & Wand, 1987). Backbone assignments were obtained using the following standard set of 2D and 3D heteronuclear spectra: ^{1}H-^{15}N HSQC, HNCACB, CBCA(CO)NH, HACACO, HNCO, CCCONH, and ^{1}H-^{13}C HSQC. Additional assignments were made using 2D TOCSY and DQF-COSY spectra. A set of distance constraints were derived from 2D NOESY spectra recorded from a 3.0 mM Pan3 zinc finger domain sample with a mixing time of 150 ms. Hydrogen bond constraints were included for a number of backbone amide protons whose signals were still detected after 10 mins in a 2D ^{1}H-^{15}N HSQC spectrum recorded in D_2O at 278 K (pH 5.0). For hydrogen bond partners, two distance constraints were used where the distance $^{(D)}H$-$O^{(A)}$ corresponded to 1.5–2.5 Å and $^{(D)}N$-$O^{(A)}$ to 2.5-3.5 Å. Torsional angle constraints were obtained from an analysis of C', N, C_α H_α and C_β chemical shifts using the program TALOS (Cornilescu *et al*, 1999).

The stereospecific assignments of Hβ resonances determined from DQF-COSY and HNHB spectra were confirmed by analyzing the initial ensemble of structures. Stereospecific assignments of Hγ and Hδ resonances of Val and Leu residues, respectively, were assigned using a fractionally ^{13}C-labelled protein sample (Neri *et al*, 1989). Stereospecific assignments were identified for resolved resonances when the side-chain atoms were sufficiently well-defined in the ensemble of structures. The three-dimensional structures of the Pan3 zinc finger domain were calculated using the standard torsion angle dynamics-simulated annealing protocol in the program CNS 1.2 (Brunger, 2007). Structures were accepted where no distance violation was greater than 0.25 Å and no dihedral angle violations > 5°.

NMR chemical shift mapping

The site of interaction with polyA was determined by monitoring the changes in the 2D ^{1}H-^{15}N-HSQC spectra of the scPan3 zinc finger domain upon the addition of a 15-mer polyA. RNA binding altered the NMR spectrum of the domain (Supplementary Fig. 4). A number of peaks change chemical shift, others decrease in intensity and one disappears (Arg15). NMR-monitored titrations indicated that the peaks that change chemical shift are in the fast exchange regime relative to the chemical shift time scale. The peaks that disappear presumably undergo larger changes in chemical shifts upon binding and are in the intermediate regime. To determine the affinity for RNA, the observed

chemical shift perturbations for each residue (δ_{obs}) were fit using Graphpad Prism to:

$$\delta_{obs} = \frac{\delta_{max}[R_T]}{[R_T] + K_d}$$

where δ_{max} is the maximal shift, $[R_T]$ is the total concentration of RNA and K_d is the apparent dissociation constant.

Pan3 structure determination

Following initial screening of sparse-matrix crystallization conditions, crystals were optimized through micro seeding from initial hits. Optimized crystals were grown in 0.1 M MES pH 6.5, 7% (w/v) PEG 8000, 19% (v/v) glycerol, 0.2 M $MgCl_2$. Crystals of the selenomethionine-derivatized protein were grown under identical conditions except that the solutions were supplemented with 5 mM TCEP. For cryoprotection prior to flash-cooling in liquid nitrogen, the crystals were transferred briefly to a drop containing the crystallization solution plus 1/5 volume glycerol. Diffraction data were collected on beamline I03 at the Diamond Light Source (Didcot, U.K.) from a single crystal at the peak wavelength for selenium identified through a fluorescence scan. A fine phi-slicing data collection strategy was employed with the final dataset comprising 5,000 frames. The crystals diffracted to 2.42 Å resolution and had *P*1 symmetry with eight Pan3 chains arranged into four homodimers.

Reflections were indexed and integrated using the three-dimensional profile-fitting method as implemented in *XDS* (Kabsch, 2010). Reflections were merged and averages intensities obtained using *AIMLESS* (Evans & Murshudov, 2013), keeping the Friedel intensities separate in the resolution range of 52.2 – 2.42 Å. The final set of structure factors with anomalous scattering was used to find 72 selenium sites using *SHELXD* (Usón & Sheldrick, 1999). The heavy atom sites and the set of structure factor amplitudes were then used for phasing using *SHARP* (Bricogne *et al*, 2003). After several cycles of phasing and refinement of the heavy atom positions, followed by solvent flattening as implemented in SOLOMON (Abrahams & Leslie, 1996), an electron density map of sufficient quality was generated to enable automated model building and phase improvement using *BUCCANEER* (Cowtan, 2006). The initial model generated was then subjected to several rounds of maximum-likelihood-based restrained refinement using *BUSTER-TNT* (Bricogne *et al*, 2011) together with manual adjustment in *COOT* (Emsley *et al*, 2010), after which a final model was generated using the *PHENIX* suite (Adams *et al*, 2010). Refinement strategies utilized non-crystallographic symmetry restraints in the form of Local Structure Similarity Restraints (LSSR) function in *BUSTER-TNT* (Bricogne *et al*, 2011) and in-torsional space in *PHENIX* as well as TLS (Translation/Libration/Screw) parameterization to model anisotropic displacement. The final model comprising four dimers of *ct*Pan3 in the asymmetric unit was refined to 2.42 Å resolution

(Supplementary Table S2) and had R_{work}/R_{free} values of 0.18/0.23 and excellent geometry and a MolProbity (Chen *et al*, 2010) score of 1.30 (100th percentile).

Pan2–Pan3 complex structure determination

Following initial screening of sparse-matrix crystallization conditions, crystals of the *ct*Pan2 PID–Pan3 PKC complex were observed in several conditions. Crystals that had $P2_1$ symmetry were obtained from 0.1 M MES/imidazole pH 6.5, 10% (w/v) PEG4000, 20% (v/v) glycerol and 20 mM each of NaGlu, DL-Ala, Gly, DL-Lys and DL-Ser and were cryoprotected in the crystallization solution plus 1/5 volume glycerol. Diffraction data were collected from a single crystal on beamline ID14-4 at the European Synchrotron Radiation Facility (ESRF, Grenoble, France). Reflections were indexed and integrated using three-dimensional profile-fitting as implemented in *XDS* (Kabsch, 2010). Reflections were merged and intensities computed to 2.59 Å resolution using *AIMLESS* (Evans & Murshudov, 2013). Calculation of the Matthews coefficient from the unit cell parameters and the molecular weight of the *ct*Pan2-Pan3 complex indicated that there were two dimers in the asymmetric unit. Phases were readily obtained by molecular replacement using the *PHENIX* suite (Adams *et al*, 2010) using the coordinates of the *ct*Pan3 dimer as a search model. Continuous difference density that could not be explained by the *ct*Pan3 structure alone was clearly visible in the F_0-F_C electron density maps computed using the initial molecular replacement phases. The phase information and the

map quality were sufficiently high to enable automated tracing of the *ct*Pan2 chain using *BUCCANEER* (Cowtan, 2006). This was followed by iterative rounds of manual rebuilding in *COOT* (Emsley *et al*, 2010) interspersed with reciprocal-space refinement carried out using the *PHENIX* suite (Adams *et al*, 2010) using non-crystallographic symmetry restraints in torsional space together with TLS parameterization to model anisotropic displacement. The final model, comprising two dimers of *ct*Pan3 and two chains of *ct*Pan2 in the asymmetric unit, was refined to 2.59 Å resolution and had R_{work}/R_{free} values of 0.18/0.22 (Supplementary Table S3) with excellent geometry and a MolProbity (Chen *et al*, 2010) score of 1.09 (100th percentile). Most residues of the *ct*Pan3 dimer were visible in the electron density, except for two surface loops in the kinase domain (residues 260–264 in chain B and 260–263 in chain D, residues 354–362 in chain A, 354–358 in chain B, 355–362 in chain C and 354–360 in chain D), one surface loop in the CTD (residues 543–554 in chain A, 544–546 in chain B and D and 543–553 in chain C) as well as N- and C-terminal extensions (residues 205–206 in chains A, B and D and 205–209 in chain D, residues 631–640 in chain A, 632–640 in chain B and D and 633–640 in chain C). Figures were prepared using PyMOL (The PyMOL Molecular Graphics System, Version 1.5.0.4 Schrödinger, LLC).

Pull-down assays

N-terminally StrepII-tagged *ct*Pan2 truncation or PID-deletion constructs were co-expressed with *ct*Pan3 PKC in *S. cerevisiae* BCY123 cells (see above:

Protein expression and purification). Cells were lysed in 50 mM Tris pH 8.0, 500 mM NaCl, 2 mM TCEP, EDTA-free protease inhibitor tablets (Roche) and DNaseI by bead beating using glass beads for 10 min at 4 °C. Cleared cell lysates were incubated with Streptactin Sepharose (GE Healthcare) for 1 hour at 4 °C, washed 5 times in lysis buffer and bound proteins eluted with 5 mM desthiobiotin in lysis buffer. Elution fractions were analyzed on a 4-12% NuPage Bis-Tris precast gel (Life Technologies).

N-terminally GST-tagged *ct*Pan3 PKC was co-expressed with *ct*Pan2 WD40-PID domain constructs in *Escherichia coli* BL21 (DE3) cells. Cells were lysed in 50 mM Tris pH 7.4, 500 mM NaCl, 2 mM TCEP, EDTA-free protease inhibitor tablets (Roche) and DNaseI by sonication. Cleared lysates were incubated with glutathione Sepharose for 1 hour at 4 °C and washed 5 times with lysis buffer. Beads were incubated with TEV protease for 2 hour at room temperature. TEV elution fractions were analyzed on a 10% NuPage Bis-Tris precast gel (Life Technologies).

N-terminally GST-tagged *ct*Pan2 PID constructs were expressed in *Escherichia coli* BL21 (DE3) cells. Cells were lysed in 50 mM Tris pH 7.4, 500 mM NaCl, 2 mM TCEP, EDTA-free protease inhibitor tablets (Roche) and DNaseI by sonication. Cleared lysates were incubated with glutathione Sepharose for 1 hour at 4 °C and washed 5 times with lysis buffer. Beads were incubated 1 hour at room temperature with purified *ct*Pan3 PKC and washed 5 times with lysis

buffer. Bound proteins were analyzed on a 10% NuPage Bis-Tris precast gel (Life Technologies).

Surface plasmon resonance

Surface Plasmon Resonance (SPR) measurements were made using a BIAcore T200 instrument (GE Healthcare) at a flow rate of 30 µl min^{-1} in 20 mM Tris-HCl, pH 7.4, 500 mM NaCl, 0.005 % (v/v) Tween-20 at 25 ˚C. GST-tagged *ct*Pan2 (343-458), or recombinant GST on the reference channel, were captured by an anti-GST antibody-coated CM5 sensor chip (GE Healthcare) prepared according to the supplied instructions. A series of concentrations of *ct*Pan3 PKC (116 nM, 58 nM, 29 nM, 14.5 nM, 7.25 nM) was injected for 120 s and dissociation monitored for 600 s. The sensor surface was regenerated after each injection with a 1 min injection of 10 mM glycine, pH 2.1.

Isothermal titration calorimetry

Measurements were performed using an ITC 200 instrument (GE Healthcare) in 20 mM Tris-HCl, pH 7.4, 500 mM NaCl, at 25°C. 13.75 µM *ct*Pan3 PKC was titrated into 1.6 µM GST-*ct*Pan2 (343-458) protein in a series of 19 × 2 µl injections, preceded by a single 0.5 µl pre-injection. Heat from the pre-injection was not used during fitting. Data were analysed in the Origin software package provided by the manufacturer and fitted to a single-site binding model.

Measurements were corrected using control ITC experiments in which the *ct*Pan3 PKC was injected into buffer only.

SUPPLEMENTARY REFERENCES

Abrahams JP & Leslie AG (1996) Methods used in the structure determination of bovine mitochondrial F1 ATPase. *Acta Crystallogr. D Biol. Crystallogr.* **52:** 30–42

Adams PD, Afonine PV, Bunkóczi G, Chen VB, Davis IW, Echols N, Headd JJ, Hung L-W, Kapral GJ, Grosse-Kunstleve RW, McCoy AJ, Moriarty NW, Oeffner R, Read RJ, Richardson DC, Richardson JS, Terwilliger TC & Zwart PH (2010) PHENIX: a comprehensive Python-based system for macromolecular structure solution. *Acta Crystallogr. D Biol. Crystallogr.* **66:** 213–221

Bax A, Ikura M, Kay LE, Barbato G & Spera S (1991) Multidimensional triple resonance NMR spectroscopy of isotopically uniformly enriched proteins: a powerful new strategy for structure determination. *Ciba Found. Symp.* **161:** 108–19 discussion 119–35

Bricogne G, Blanc E, Brandl M, Flensburg C, Keller P, Paciorek W, Roversi P, Sharff A, Smart OS, Vonrhein C & Womack TO (2011) Buster version 2.10.0.

Bricogne G, Vonrhein C, Flensburg C, Schiltz M & Paciorek W (2003) Generation, representation and flow of phase information in structure determination: recent developments in and around SHARP 2.0. *Acta Crystallogr. D Biol. Crystallogr.* **59:** 2023–2030

Brunger AT (2007) Version 1.2 of the Crystallography and NMR system. *Nat. Protoc.* **2:** 2728–2733

Chen VB, Arendall WB, Headd JJ, Keedy DA, Immormino RM, Kapral GJ, Murray LW, Richardson JS & Richardson DC (2010) MolProbity: all-atom structure validation for macromolecular crystallography. *Acta Crystallogr. D Biol. Crystallogr.* **66:** 12–21

Christianson TW, Sikorski RS, Dante M, Shero JH & Hieter P (1992) Multifunctional yeast high-copy-number shuttle vectors. *Gene* **110:** 119–122

Cornilescu G, Delaglio F & Bax A (1999) Protein backbone angle restraints from searching a database for chemical shift and sequence homology. *J. Biomol. NMR* **13:** 289–302

Cowtan K (2006) The Buccaneer software for automated model building. 1. Tracing protein chains. *Acta Crystallogr. D Biol. Crystallogr.* **62:** 1002–1011

Dodd RB, Allen MD, Brown SE, Sanderson CM, Duncan LM, Lehner PJ, Bycroft M & Read RJ (2004) Solution structure of the Kaposi's sarcoma-associated herpesvirus K3 N-terminal domain reveals a Novel E2-binding C4HC3-type RING domain. *J. Biol. Chem.* **279:** 53840–53847

Emsley P, Lohkamp B, Scott WG & Cowtan K (2010) Features and development of Coot. *Acta Crystallogr. D Biol. Crystallogr.* **66:** 486–501

Englander SW & Wand AJ (1987) Main-chain-directed strategy for the assignment of 1H NMR spectra of proteins. *Biochemistry* **26:** 5953–5958

Evans PR & Murshudov GN (2013) How good are my data and what is the resolution? *Acta Crystallographica Section D:* **69**: 1204-1213

Galej WP, Oubridge C, Newman AJ & Nagai K (2013) Crystal structure of Prp8 reveals active site cavity of the spliceosome. *Nature* **493:** 638–643

Hudson BP, Martinez-Yamout MA, Dyson HJ & Wright PE (2004) Recognition of the mRNA AU-rich element by the zinc finger domain of TIS11d. *Nat. Struct. Mol. Biol.* **11:** 257–264

Kabsch W (2010) XDS. *Acta Crystallogr. D Biol. Crystallogr.* **66:** 125–132

Kuhlmann SI, Valkov E & Stewart M (2014) Structural basis for the molecular recognition of polyadenosine RNA by Nab2 Zn fingers. *Nucleic Acids Res.* **42:** 672–680

Lai WS, Perera L, Hicks SN & Blackshear PJ (2014) Mutational and structural analysis of the tandem zinc finger domain of tristetraprolin. *J. Biol. Chem.* **289:** 565–580

Neidhardt FC, Bloch PL & Smith DF (1974) Culture medium for enterobacteria. *J. Bacteriol.* **119:** 736–747

Neri D, Szyperski T, Otting G, Senn H & Wüthrich K (1989) Stereospecific nuclear magnetic resonance assignments of the methyl groups of valine and leucine in the DNA-binding domain of the 434 repressor by biosynthetically directed fractional 13C labeling. *Biochemistry* **28:** 7510–7516

Sievers F, Wilm A, Dineen D, Gibson TJ, Karplus K, Li W, Lopez R, McWilliam H, Remmert M, Söding J, Thompson JD & Higgins DG (2011) Fast, scalable generation of high-quality protein multiple sequence alignments using Clustal Omega. *Mol. Syst. Biol.* **7:** 539

Teplova M & Patel DJ (2008) Structural insights into RNA recognition by the alternative-splicing regulator muscleblind-like MBNL1. *Nat. Struct. Mol. Biol.* **15:** 1343–1351

Usón I & Sheldrick GM (1999) Advances in direct methods for protein crystallography. *Curr. Opin. Struct. Biol.* **9:** 643–648

Wagenbach M, O'Rourke K, Vitez L, Wieczorek A, Hoffman S, Durfee S, Tedesco J & Stetler G (1991) Synthesis of wild type and mutant human hemoglobins in Saccharomyces cerevisiae. *Bio/technology (Nature Publishing Company)* **9:** 57–61

Waterhouse AM, Procter JB, Martin DMA, Clamp M & Barton GJ (2009) Jalview Version 2--a multiple sequence alignment editor and analysis workbench. *Bioinformatics* **25:** 1189–1191
