# Supplementary material for: Structural basis for Pan3 binding to Pan2 and its function in mRNA recruitment and deadenylation
Source: EMBO J. 2014 May 29;33(14):1514–26. doi: 10.15252/embj.201488373 (PMC4158885; doi:10.15252/embj.201488373)
Supplement: Supplementary file 13 [file embj0033-1514-sd13.pdf]

THE EMBO JOURNAL

Manuscript EMBO-2014-88373

Structural basis for Pan3 binding to Pan2 and its function in mRNA recruitment and deadenylation

Jana Wolf, Eugene Valko, Mark D. Allen, Birthe Meineke, Yuliya Gordiyenko, Stephen H. McLaughlin, Tayla M. Olsen, Carol V. Robinson, Mark Bycroft, Murray Stewart and Lori A. Passmore

Corresponding author: Lori A. Passmore, MRC, Laboratory of Molecular Biology

Review timeline:

Submission date:	03 March 2014
Editorial Decision:	21 March 2014
Revision received:	25 April 2014
Accepted:	03 May 2014

Editor: Hartmut Vodermaier

Transaction Report:

(Note: With the exception of the correction of typographical or spelling errors that could be a source of ambiguity, letters and reports are not edited. The original formatting of letters and referee reports may not be reflected in this compilation.)

1st Editorial Decision 21 March 2014

Thank you for submitting your manuscript on Pan3-Pan2 cooperation and complex structure to The EMBO Journal. We have now received the attached comments of four expert referees, and I am pleased to inform you that all of them consider this work important and in principle suitable for publication, pending satisfactory addressing of several specific issues raised. We shall therefore be happy to consider a revised version of the manuscript further for publication.

Please keep in mind that it is our policy to allow only a single round of major revision and that it is therefore important to carefully respond to all points at this stage. We generally allow three months as standard revision time, and it is our policy that competing manuscripts published during this period will have no negative impact on our final assessment of your revised study; should you foresee a problem in meeting this three-month deadline, please let me know in advance and we could discuss the possibility of an extension.

Thank you again for the opportunity to consider this work, and please do not hesitate to contact me in case you should have any additional question regarding this decision or the reports. I look forward to your revision!

Referee #1:

Pan2/Pan3 is one of two universally conserved mRNA deadenylases. Whereas the catalytic activity resides in Pan2, the role of Pan3 is not well understood except that it mediates an interaction of the complex with the poly(A) binding protein (Pab1). The authors report that a single Pan2 subunit

© European Molecular Biology Organization

associates with a Pan3 homodimer. They analyze the structural basis of the interaction by solving the crystal structure of a Pan2 peptide bound to the Pan3 dimer. In addition to binding Pab1, Pan3 can also bind RNA directly with a zinc finger, which recognizes poly(A) specifically, and with its C-terminal domain, which binds non-specifically. The structure of the zinc finger is also presented. In agreement with direct RNA binding by Pan3, the authors show that the complex can degrade poly(A) in the absence of Pab1 with reasonably high efficiency. As Pan2 has very low affinity for RNA, it is obviously the function of Pan3 to mediate substrate binding.
Overall, the paper reports a number of interesting new aspects regarding the structure of Pan and its substrate recognition, and the claims are supported by good data. I recommend that the paper be published after some details have been taken care of.

Detailed comments:

1. Nuclease assays: The authors report protein concentrations in molar units, but substrate amounts in mass units (p. 24). Please convert to molar concentration. My crude estimate is an RNA concentration of 300 nM. This would be a significant excess of substrate over enzyme, i. e. perfect conditions.

2. The authors report enzymatic and binding activities for a number of different proteins. They should provide some evidence that the activities are not due to contaminations. They have to consider that E. coli has several potent 3' exonucleases, and in binding assays with proteins having micromolar affinities even a very low level contamination by a nanomolar binder will be detectable. (This is obviously not true for the NMR experiments.) For the scPan complex, they show that the zinc finger deletion reduces activity, and they should show (or at least state) that the active site mutant they used for shift assays indeed has no activity; this would make contaminations as a source of the activity unlikely. Another useful type of experiment would be to check that the peak of the activity examined co-elutes from columns together with the peak of the protein. In this respect, Fig. S1A, which documents the purity of protein preps, is lacking ct Pan3 PKC (which is used for the binding assays in Fig. 4).

3. Regarding the Pab-independent activity, the authors might wish to discuss two details: First, Lowell et al. already described conditions under which they saw Pab1-independent activity. I also seem to remember that their standard reaction conditions were somewhat strange, I believe they used quite low ionic strength. Anyway, since the requirement for Pab1 seems to depend on conditions, the question of whether Pan depends on Pab in vivo might be worth a comment. Second, data in Lowell et al. showed that even under conditions of Pab-independent activity, Pan was poly(A)-specific. This agrees with data reported in the current manuscript and should be mentioned.

4. The authors use NMR to demonstrate an interaction of the Pan3 zinc finger with poly(A). It is unclear to which equation the data in Fig. S3B were fitted.

5. Please report KD values for the binding data in Figs. 3 and 4. The difference in affinity between the polyadenylated and the non-polyadenylated RNA is only about threefold. This would suggest that there may be additional contributions to substrate specificity, for example of the active site.

6. The authors argue that Pan2 by itself is inactive because it has a low affinity for RNA, and that the role of Pan3 is limited to facilitating the interaction of RNA with the complex. A relatively simple method to examine this idea would be a steady-state Michaelis-Menten analysis. The authors could either use homogeneously labeled poly(A) for this purpose or detect the AMP released by a coupled assay. I would not make this type of experiment a condition for acceptance of the paper, but it would certainly improve the manuscript.

7. Fig. 5A: I could not find details of the Pan2 constructs used for the pull-down. Please mention in the figure legend where they can be found (amino acid numbering of expressed fragments).

8. Fig. 5C and D: The figure shows ATP bound to Pan3. I do not think this is discussed or even mentioned in the paper.

9. Fig. S5D and E, and text on p. 16 - 17: I do not agree with the interpretation of this experiment. Fig. S5D shows weaker Pan3 binding with amino acids 1 - 342 of Pan2 and good binding with aa 1 -

352. S5E shows good Pan3 binding with aa 353 - 458 of Pan2. As there is no overlap between the binding fragments, there is no region in the PID that is absolutely required for association with Pan3. Thus, I do not see the basis for claiming that residues 343 - 406 are important for binding. With respect to the presentation, remind people which amino acids you are talking about when you say '53 out of the 142 residues (p. 16).

Minor points:

The authors repeatedly mention that poly(A) shortening can regulate translation. While there are clear-cut examples for this type of regulation, the recent paper by Subtelny (cited in the manuscript) suggests that this may be limited to specific cell types. The authors might wish to modify their statement.

Abstract and p. 14, line 5 from the bottom: The authors use the word 'albeit' inappropriately. (My opinion was confirmed by a native speaker.) In the context, 'although' would be appropriate.

The concept of 'terminal deadenylation' (p. 3 and Discussion) is not discussed correctly. The term 'terminal deadenylation' has been used (by Tucker et al., 2001) for the very last phase of deadenylation, removal of the last dozen A residues or so. Published data certainly show that the CR4-NOT complex is involved throughout most of the deadenlyation reaction; its role is not limited to the very last phase. My impression of the Tucker experiments is that most of the deadenylation reaction can be carried out by either Pan or CCR4-NOT.
The first proposal that the Pan complex acts very early was in the paper by Brown et al. (1998); this should be cited on p. 3, line four from the bottom.

In the final paragraph of the Introduction (p. 5), the authors raise the question how many exonuclease subunits are present in the complex. As the remaining part of this paragraph is a summary of the results, it would be appropriate to answer the question here.

p. 24: 'CYC1 3' UTR' - which nucleotides?

I would be interested to know why the authors treated their linearized transcription template with mung bean nuclease (p. 24).

Fig. S4D: Counting from amino acid position 343, I believe the number 353 should be moved one position to the left (to the R). Then, the numbering of the conserved F and W residues would also agree between text (p. 15) and figure.

Referee #2:

This is an interesting manuscript that uses biochemical and biophysical assays to significantly extend our understanding of the Pan2-Pan3 deadenylase. While there have been a number of papers published over the last ~10 years on the Pan2/3 complex, this one in my opinion paints perhaps the clearest picture of the enzyme and its cofactor to date. Key points raised by this study include insights into RNA binding properties of Pan3 and Pan2, as well as a 2.6A structure of the Pan2-Pan3 complex which demonstrates an extensive interaction domain. The data generally support the conclusions that are drawn and the study should have impact in the field. I only have two minor suggestions to improve the study/its presentation:

1. As a failsafe control for Fig.1/FigS1, Pab1 should be added to the RNA substrate on its own to demonstrate that it did not contain contaminating nuclease activity which might account for the 4X stimulation in decay. Admittedly, the data that are presented do look very clean, but at least the statement of this negative control in the text would make the experimental design more optimal.
2. Fig 4: While the conclusion drawn from these data is likely very valid, since every protein that is analyzed is showing some binding, the addition of a negative control to the experiment would clearly establish the relevance of the low affinity binding that is reported.

P.S. Don't forget to add the PDB accession numbers on Pg. 26 when available.

Referee #3:

In this manuscript, Passmore and colleagues studied the structure and function of the Pan2-Pan3 deadenylation complex. They show that Pan3 contains a previously uncharacterized zinc finger, which binds RNA with low affinity, and contributes to Pan3's overall affinity for RNA. Building on a recently published structure of the pseudokinase domain of Pan3, the authors report here the structure of a fragment (PID) of Pan2 bounds to Pan3. The structure shows an unusual wrapping of the Pan3 coiled-coil by the Pan2 sequence. l By mass spectrometry, they confirm the stoichiometry observed in the crystal is the same as observed with the full-length yeast Pan2/Pan3.

Overall this manuscript is an important and timely addition to understanding of mRNA deadenylation. There is little to criticize - the seven figures in the manuscript are supported by six multi-panel supplementary figures and three tables. The data are of high quality. The text is clear.

Minor issues:

The specificity of the zinc finger for polyA does not appear to be as strong as the authors suggest. Suppl Fig 3A shows some changes for polyG. Perhaps it would be more accurate to say polyC and polyU generated negligible changes while polyG generated small changes (pg 9, top).

In Fig 4A, it would help if the colors of the curves were more different. It is impossible to distinguish between the A15 and C15 curves.

PID should be added to the list of abbreviations.

In Suppl Fig 5, was the Kd calculated by SPR and ITC using the concentration of Pan3 dimers? The legend to panel A says "concentrations of ctPan3 PKC" while panel B says "ctPan3 PKC dimer".

There must be a typo in Table S3 since the space group P21 should have two 90{degree sign} angles.

The title might be changed to "Structural basis for Pan3 binding to Pan2 and its function in mRNA recruitment and deadenylation"

Referee #4:

The Pan2-Pan3 deadenylase complex functions to shorten the poly(A) tails of mRNA in general and miRNA-mediated mRNA decay pathways. In this complex, Pan2 acts as an exonuclease while PAN3 binds PAN2 to facilitate efficient deadenylation. The previous structure of PAN3 shows that PAN3 forms an asymmetric homodimer with dimerization mediated by an intertwined coiled coil linking the pseudokinase and C-terminal domains. But how PAN3 binds Pan2 and enhances mRNA deadenylation remains elusive. In this manuscript, Wolf et al. showed that PAN3 binds RNA directly through both its pseudokinase/C-terminal domain and an N-terminal zinc finger whereas Pan2 is unable to bind RNA. The N-terminal zinc finger of PAN3 binds ploy(A) RNA specifically while its pseudokinase/C-terminal domain has no RNA binding preference. More importantly, they identified that Pan3 binds to the linker region of Pan2 connecting its N-terminal WD40 domain to the C-terminal exonuclease domain with a 2:1 stoichiometry. They further showed that the Pan2 linker region mediates the Pan2 binding to Pan3 with high affinity through structural determination of a Pan3 homodimer in complex with the Pan2 linker region. These results suggest that Pan3 supplies Pan2 with substrate RNA through its direct interaction with Pan2 for efficient deadenylation. The results presented in this manuscript revealed the molecular basis of Pan2-Pan3 interaction and are important contributions towards understanding the mechanism governing mRNA deadenylation by the Pan2-Pan3 complex. The manuscript is well written and is very pleasant to read. My only suggestion is that it might be worthwhile examining the effect of the Pan2 PID deletions on the deadenylation activity of Pan2-Pan3.

1st Revision - authors' response 25 April 2014

Point-by-point response to referees' comments

Referee #1:

Pan2/Pan3 is one of two universally conserved mRNA deadenylases. Whereas the catalytic activity resides in Pan2, the role of Pan3 is not well understood except that it mediates an interaction of the complex with the poly(A) binding protein (Pab1). The authors report that a single Pan2 subunit associates with a Pan3 homodimer. They analyze the structural basis of the interaction by solving the crystal structure of a Pan2 peptide bound to the Pan3 dimer. In addition to binding Pab1, Pan3 can also bind RNA directly with a zinc finger, which recognizes poly(A) specifically, and with its C-terminal domain, which binds non-specifically. The structure of the zinc finger is also presented. In agreement with direct RNA binding by Pan3, the authors show that the complex can degrade poly(A) in the absence of Pab1 with reasonably high efficiency. As Pan2 has very low affinity for RNA, it is obviously the function of Pan3 to mediate substrate binding.
Overall, the paper reports a number of interesting new aspects regarding the structure of Pan and its substrate recognition, and the claims are supported by good data. I recommend that the paper be published after some details have been taken care of.

We thank the referee their constructive comments and for identifying areas in which the manuscript could be strengthened. We have modified the manuscript and performed the control experiments along the lines suggested.

Detailed comments:

1. Nuclease assays: The authors report protein concentrations in molar units, but substrate amounts in mass units (p. 24). Please convert to molar concentration. My crude estimate is an RNA concentration of 300 nM. This would be a significant excess of substrate over enzyme, i. e. perfect conditions.

The referee is correct and we now give all concentrations in molarity. We have converted the substrate amount into molar concentration: We used 180 nM RNA which is ~36-fold excess over the enzyme concentration. This information is now included in the Materials and Methods in the main paper (page 26).

2. The authors report enzymatic and binding activities for a number of different proteins. They should provide some evidence that the activities are not due to contaminations. They have to consider that E. coli has several potent 3' exonucleases, and in binding assays with proteins having micromolar affinities even a very low level contamination by a nanomolar binder will be detectable. (This is obviously not true for the NMR experiments.) For the scPan complex, they show that the zinc finger deletion reduces activity, and they should show (or at least state) that the active site mutant they used for shift assays indeed has no activity; this would make contaminations as a source of the activity unlikely. Another useful type of experiment would be to check that the peak of the activity examined co-elutes from columns together with the peak of the protein.

We have included additional controls to address the issue of contaminating nucleases and binding proteins in our assays. (We note that none of the proteins used in our deadenylation assays were expressed in *E. coli* – they were all expressed in *S. cerevisiae* – but yeast could also be a source of contamination). We have now performed deadenylation assays with the different active site mutants (*sc*Pan2-E912A and *ct*Pan2-E899A) as well as Pab1 alone and show that these are inactive (Supplementary Fig S2). Thus, the deadenylation activity we observe is not due to contaminating proteins.

In addition, in fluorescence polarization assays (Fig 4A-B), we used 20 nM RNA and micromolar concentrations of protein. If exonuclease activity was due to contamination, we would expect degradation of the RNA in these experiments. This is not the case. In summary, it is unlikely that the activity we observe in our deadenylation assay is due to contamination with co-purifying nucleases.

We also provide an additional control for our binding experiments: In Fig 4A and 4B, we show that purified GST (like isolated *ct*Pan2) does not alter the fluorescence polarization of RNA substrates, whereas *ct*Pan3PKC or *ct*Pan2–Pan3PKC do. GST is a good control for contaminants in the purification procedure since *ct*Pan3PKC was purified as a GST-fusion protein.

In this respect, Fig. S1A, which documents the purity of protein preps, is lacking ct Pan3 PKC (which is used for the binding assays in Fig. 4).

We thank the referee for pointing this out and now include a gel of *ct*Pan3PKC in Supplementary Fig S1A.

3. Regarding the Pab-independent activity, the authors might wish to discuss two details: First, Lowell et al. already described conditions under which they saw Pab1-independent activity. I also seem to remember that their standard reaction conditions were somewhat strange, I believe they used quite low ionic strength. Anyway, since the requirement for Pab1 seems to depend on conditions, the question of whether Pan depends on Pab in vivo might be worth a comment.

Second, data in Lowell et al. showed that even under conditions of Pab-independent activity, Pan was poly(A)-specific. This agrees with data reported in the current manuscript and should be mentioned.

The standard reaction conditions used by Lowell *et al.* indeed had a very low ionic strength with only 2.5 mM K acetate. Moreover, the RNA substrates that Lowell *et al.* used to demonstrate dependence on Pab1 had long (A_{100}) polyA tails with 6-7 nonadenosine nucleotides at their 3' end.

Lowell *et al.* showed that Pan is active in the absence of Pab1 under two conditions: 1) in the presence of spermidine, and 2) on short (A_{25}) polyA tails. Notably the RNA substrate used for these experiments differed from their standard RNA substrate as it contained adenosines at the 3' end. This could also explain the difference in Pab1-dependence. In our experiments, we used a RNA substrate with only adenosines at the 3' end (A_{80}), as we believe this is the most physiologically relevant substrate.

The data presented by Lowell *et al.* suggests that Pan has a preference for polyA but is still able to remove other nucleotides in the presence of Pab1. The Pab1-independent activity of Pan was specific for polyA. Thus, these data are consistent with our observations.

It is unclear whether Pab1 is required for Pan2–3 activity *in vivo*. We now discuss this and the Pab1-independent activity (that is dependent on the conditions used, Lowell *et al*) in the Discussion on page 23 as follows:

"…It is conceivable that Pan2–Pan3 could function independently of Pab1 *in vivo*. Pab1-independent activity of Pan2–Pan3 was observed previously in very low ionic strength conditions and in the presence of spermidine (Lowell *et al*, 1992). Importantly, Pan2–Pan3 retains polyA specificity in the absence of Pab1 (Fig 1B-C; Lowell *et al*, 1992)…."

4. The authors use NMR to demonstrate an interaction of the Pan3 zinc finger with poly(A). It is unclear to which equation the data in Fig. S3B were fitted.

To determine the affinity for RNA, the observed chemical shift perturbations for each residue (δ_{obs}) were fit using Graphpad Prism to:

$$\delta_{obs} = \frac{\delta_{\max}[R_T]}{[R_T] + K_d}$$

where $\delta_{\max}$ is the maximal shift, $[R_T]$ is the total concentration of RNA and K_d is the apparent dissociation constant (now included in the "NMR chemical shift mapping" section of the

Supplementary Methods). We note that the fit of the data to this model is very good whereas fitting to more complex models was less successful.

5. Please report KD values for the binding data in Figs. 3 and 4. The difference in affinity between the polyadenylated and the non-polyadenylated RNA is only about threefold. This would suggest that there may be additional contributions to substrate specificity, for example of the active site.

We have added K_d values for the binding data in Fig 4A and 4B where we analyzed binding to fluorescently-labeled short RNAs and could accurately quantitate the results (see Figure legends).

We feel less comfortable adding K_d values to Fig 3 as we found that it is more difficult to interpret these data quantitatively. The experiments in Fig 3 are reproducible and qualitatively show that the zinc finger contributes to polyA specificity, but quantitation is sometimes variable due to the narrow linear range of the stain density. Moreover, in the Figure we plot loss of free RNA and we don't know how many binding sites are present on each of the long RNA molecules (the shifted RNA runs very close to the top of the gel). We have therefore performed an additional experiment to allow qualitative comparison of these samples:

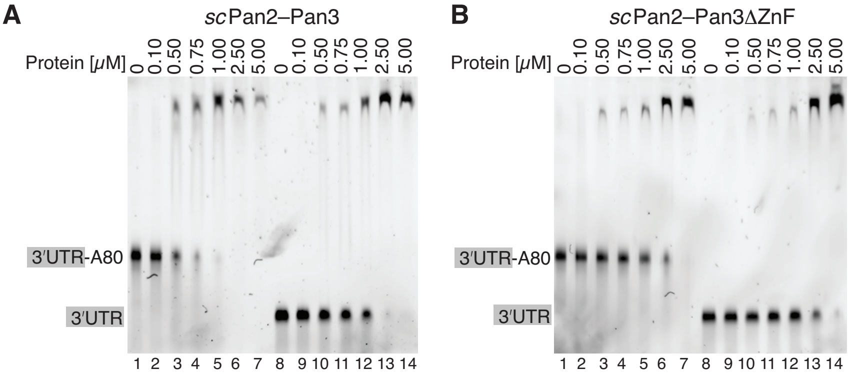

Figure R1: The zinc finger domain of *sc*Pan3 increases the affinity of the Pan2–Pan3 complex to polyA RNA. Electrophoretic mobility shift assay (EMSA) using the *CYC1* 3' UTR RNA with or without a polyA$_{80}$ tail. RNA was incubated with (A) *sc*Pan2–Pan3 or (B) *sc*Pan2–Pan3 with a deletion of the Pan3 zinc finger (*sc*Pan2–Pan3ΔZnF). Both complexes contain an active site mutation in *sc*Pan2 (E912A). Binding was analyzed by native polyacrylamide gel electrophoresis.

This figure is now included in Supplementary Fig S5 and shows the trend that we always observe for RNA binding: We consistently observed that full-length Pan2–Pan3 binds polyadenylated RNA (Fig R1A, lanes 2-7) more tightly than a 3'UTR without polyA tail (Fig R1A, lanes 9-14), but Pan2–Pan3 with the Pan3 zinc finger deleted binds both RNAs similarly (Fig R1B). We agree that there are likely additional contributions to substrate specificity and have added the following statement towards the end of the Discussion (page 24):

"Other components, including the active site of Pan2, may also contribute to polyA specificity."

6. The authors argue that Pan2 by itself is inactive because it has a low affinity for RNA, and that the role of Pan3 is limited to facilitating the interaction of RNA with the complex. A relatively simple method to examine this idea would be a steady-state Michaelis-Menten analysis. The authors could either use homogeneously labeled poly(A) for this purpose or detect the AMP released by a coupled assay. I would not make this type of experiment a condition for acceptance of the paper, but it would certainly improve the manuscript.

Although this could be a very interesting experiment, it is not clear whether Pan2–Pan3 exhibits Michaelis-Menten kinetics. Therefore, we feel a full mechanistic analysis along the lines suggested is beyond the scope of this work.

© European Molecular Biology Organization

7. Fig. 5A: I could not find details of the Pan2 constructs used for the pull-down. Please mention in the figure legend where they can be found (amino acid numbering of expressed fragments).

We have now added the amino acid numbering of the expressed fragments to the figure legend for Fig 5A and on the schematic diagram in Fig 5A.

8. Fig. 5C and D: The figure shows ATP bound to Pan3. I do not think this is discussed or even mentioned in the paper.

We had mentioned this briefly but now include more detail (pages 13-14):

"The *ct*Pan3 structure consists of an N-terminal pseudokinase domain (residues 243–498) to which MgATP is bound, a central asymmetric coiled-coil domain (residues 500-543) and a C-terminal domain (residues 556–640) (Fig 5C). Although ATP was bound to both pseudokinase domains, ATP was not added to crystallization conditions and is present in all copies in the asymmetric unit of the crystal. The pseudokinase lacks almost all catalytic residues and is predicted to be inactive (Christie *et al*, 2013)."

In the next paragraph, we mention that ATP is also bound in the structure of the Pan2–Pan3 complex. ATP binding has already been discussed extensively by Christie *et al* and so we do not feel it is necessary to discuss this aspect in greater detail.

9. Fig. S5D and E, and text on p. 16 - 17: I do not agree with the interpretation of this experiment. Fig. S5D shows weaker Pan3 binding with amino acids 1 - 342 of Pan2 and good binding with aa 1 - 352. S5E shows good Pan3 binding with aa 353 - 458 of Pan2. As there is no overlap between the binding fragments, there is no region in the PID that is absolutely required for association with Pan3. Thus, I do not see the basis for claiming that residues 343 - 406 are important for binding.

We thank the referee for flagging that our initial discussion of this point was somewhat confusing and so we have reworded this section (pages 17-18) to explain the results from the deletion analysis more fully. In Fig 5A, we show that the PID region is important for Pan3 binding. Since 1-315 does not bind well (Fig 5A) but 1-342 exhibits weak binding (Supplementary Fig S7D), the major Pan3 binding region of Pan2 should be located C-terminal to amino acid 315. Additionally, 353-458 binds well but 375-458 does not bind in the pull-down experiments (Supplementary Fig 7E). Fig 7 shows that deletion of 343-406 (but not deletion of 343-375) is required to substantially disrupt the interaction during size exclusion chromatography. Thus, although no region in the PID is absolutely required for Pan3 association, the deletion studies demonstrate that the major binding site is located within the 315-406 region and is therefore consistent with our crystal structure.

Although the Pan2 PID makes the principal contribution to Pan3 binding, there may of course be other contributions from regions outside this domain.

With respect to the presentation, remind people which amino acids you are talking about when you say '53 out of the 142 residues (p. 16).

We have added the amino acid numbering (residues 355-406).

Minor points:

The authors repeatedly mention that poly(A) shortening can regulate translation. While there are clear-cut examples for this type of regulation, the recent paper by Subtelny (cited in the manuscript) suggests that this may be limited to specific cell types. The authors might wish to modify their statement.

We have modified our statements about influence of polyA tail length on translation. Subtelny *et al.* demonstrated that there isn't any correlation between mean polyA tail length and the number of bound ribosomes. Although this demonstrates that mRNAs with longer polyA tails aren't more

efficiently translated, the polyA tail does play a role in translation. Specifically, there is a minimum polyA tail length required for translation so removal of the polyA tail is likely to repress translation.

Abstract and p. 14, line 5 from the bottom: The authors use the word 'albeit' inappropriately. (My opinion was confirmed by a native speaker.) In the context, 'although' would be appropriate.

We thank the referee for pointing this out and we have corrected these.

The concept of 'terminal deadenylation' (p. 3 and Discussion) is not discussed correctly. The term 'terminal deadenylation' has been used (by Tucker et al., 2001) for the very last phase of deadenylation, removal of the last dozen A residues or so. Published data certainly show that the CR4-NOT complex is involved throughout most of the deadenlyation reaction; its role is not limited to the very last phase. My impression of the Tucker experiments is that most of the deadenylation reaction can be carried out by either Pan or CCR4-NOT.
The first proposal that the Pan complex acts very early was in the paper by Brown et al. (1998); this should be cited on p. 3, line four from the bottom.

We agree that this was not clear. We have modified the text to clarify that Pan2–Pan3 and Ccr4–Not are functionally redundant, and that Pan2–Pan3 may play a more significant role in initial deadenylation, compared to Ccr4–Not (as first observed by Brown & Sachs, 1998 and subsequently verified by Tucker *et al*, 2001 and Yamashita *et al*, 2005). Notably, Pan2 mutants have defects in removing the final 20-26 adenosines (Tucker *et al*, 2001). We have changed the wording on page 3-4 to:

"Although these complexes appear to be at least partially functionally redundant, Pan2–Pan3 may be more efficient at initiating deadenylation, whereas Ccr4–Not may be more efficient at removing the final ~20-25 adenosines (Brown & Sachs, 1998; Tucker *et al*, 2001; Yamashita *et al*, 2005). This suggests that Pan2–Pan3 and Ccr4–Not could act sequentially."

In the final paragraph of the Introduction (p. 5), the authors raise the question how many exonuclease subunits are present in the complex. As the remaining part of this paragraph is a summary of the results, it would be appropriate to answer the question here.

We now include this information in the paragraph.

p. 24: 'CYC1 3' UTR' - which nucleotides?

We used the entire *CYC1* 3' UTR, which is 169 nucleotides downstream of the stop codon. This is now stated in the Materials and Methods (page 25).

I would be interested to know why the authors treated their linearized transcription template with mung bean nuclease (p. 24).

The DNA sequence of the 3' end of our dsDNA template is:
…AAAATTCAAGAGACC -3'
…TTTTAAGTTCTCTGG -5'
where the Bsa*I* recognition site is highlighted in red. Cleavage of the DNA template leaves a 5' overhang:
…AAAA -3'
…TTTTAAGT -5'
We used Mung bean nuclease to removes the 5' overhang but this probably wasn't necessary.

Fig. S4D: Counting from amino acid position 343, I believe the number 353 should be moved one position to the left (to the R). Then, the numbering of the conserved F and W residues would also agree between text (p. 15) and figure.

We thank the referee for pointing out this error. We have corrected it.

Referee #2:

This is an interesting manuscript that uses biochemical and biophysical assays to significantly extend our understanding of the Pan2-Pan3 deadenylase. While there have been a number of papers published over the last ~10 years on the Pan2/3 complex, this one in my opinion paints perhaps the clearest picture of the enzyme and its cofactor to date. Key points raised by this study include insights into RNA binding properties of Pan3 and Pan2, as well as a 2.6A structure of the Pan2-Pan3 complex, which demonstrates an extensive interaction domain. The data generally support the conclusions that are drawn and the study should have impact in the field.

I only have two minor suggestions to improve the study/its presentation:
1. As a failsafe control for Fig.1/FigS1, Pab1 should be added to the RNA substrate on its own to demonstrate that it did not contain contaminating nuclease activity, which might account for the 4X stimulation in decay. Admittedly, the data that are presented do look very clean, but at least the statement of this negative control in the text would make the experimental design more optimal.

We were pleased to receive the referee's very positive assessment of our work and thank them for their helpful and constructive comments. We now include a new supplementary figure (Fig S2) in which we test the catalytic mutants of Pan2 as well as isolated Pab1 for contaminating exonuclease activities. These new experiments demonstrate that there is no significant contaminating deadenylase activity.

2. Fig 4: While the conclusion drawn from these data is likely very valid, since every protein that is analyzed is showing some binding, the addition of a negative control to the experiment would clearly establish the relevance of the low affinity binding that is reported.

We now include a negative control for RNA binding in Fig 4A and 4B. Purified GST does not substantially bind RNA – this is an appropriate control since Pan3 PKC was purified as a GST fusion protein (although the GST was removed from Pan3 by proteolysis during purification). We also note that isolated *ct*Pan2 does not significantly bind to RNA so it also acts as a negative control.

P.S. Don't forget to add the PDB accession numbers on Pg. 26 when available.

We now include PDB accession numbers.

Referee #3:

In this manuscript, Passmore and colleagues studied the structure and function of the Pan2-Pan3 deadenylation complex. They show that Pan3 contains a previously uncharacterized zinc finger, which binds RNA with low affinity, and contributes to Pan3's overall affinity for RNA. Building on a recently published structure of the pseudokinase domain of Pan3, the authors report here the structure of a fragment (PID) of Pan2 bounds to Pan3. The structure shows an unusual wrapping of the Pan3 coiled-coil by the Pan2 sequence. By mass spectrometry, they confirm the stoichiometry observed in the crystal is the same as observed with the full-length yeast Pan2/Pan3.

Overall this manuscript is an important and timely addition to understanding of mRNA deadenylation. There is little to criticize - the seven figures in the manuscript are supported by six multi-panel supplementary figures and three tables. The data are of high quality. The text is clear.

We thank the referee for helpful and insightful comments and have modified the manuscript along the lines suggested.

Minor issues:

The specificity of the zinc finger for polyA does not appear to be as strong as the authors suggest. Suppl Fig 3A shows some changes for polyG. Perhaps it would be more accurate to say polyC and polyU generated negligible changes while polyG generated small changes (pg 9, top).

We have amended the text as suggested.

In Fig 4A, it would help if the colors of the curves were more different. It is impossible to distinguish between the A15 and C15 curves.

We have changed Fig 4A and 4B – instead of normalizing the curves to percentage change in fluorescence polarization, we show background corrected curves. This accentuates the larger magnitude of change for G15 (which we attribute to possible quadruplex formation of free G15). The colors in Fig 4A are adjusted.

PID should be added to the list of abbreviations.

We have added PID to the abbreviations.

In Suppl Fig 5, was the Kd calculated by SPR and ITC using the concentration of Pan3 dimers? The legend to panel A says "concentrations of ctPan3 PKC" while panel B says "ctPan3 PKC dimer".
The K_d was calculated using the concentration of Pan3 dimers. We have amended the figure legend.

There must be a typo in Table S3 since the space group P21 should have two 90{degree sign} angles.

We thank the referee for noticing this error. We have corrected it.

The title might be changed to "Structural basis for Pan3 binding to Pan2 and its function in mRNA recruitment and deadenylation"

We have changed the title as suggested.

Referee #4:

The Pan2-Pan3 deadenylase complex functions to shorten the poly(A) tails of mRNA in general and miRNA-mediated mRNA decay pathways. In this complex, Pan2 acts as an exonuclease while PAN3 binds PAN2 to facilitate efficient deadenylation. The previous structure of PAN3 shows that PAN3 forms an asymmetric homodimer with dimerization mediated by an intertwined coiled coil linking the pseudokinase and C-terminal domains. But how PAN3 binds Pan2 and enhances mRNA deadenylation remains elusive. In this manuscript, Wolf et al. showed that PAN3 binds RNA directly through both its pseudokinase/C-terminal domain and an N-terminal zinc finger whereas Pan2 is unable to bind RNA. The N-terminal zinc finger of PAN3 binds ploy(A) RNA specifically while its pseudokinase/C-terminal domain has no RNA binding preference. More importantly, they identified that Pan3 binds to the linker region of Pan2 connecting its N-terminal WD40 domain to the C-terminal exonuclease domain with a 2:1 stoichiometry. They further showed that the Pan2 linker region mediates the Pan2 binding to Pan3 with high affinity through structural determination of a Pan3 homodimer in complex with the Pan2 linker region. These results suggest that Pan3 supplies Pan2 with substrate RNA through its direct interaction with Pan2 for efficient deadenylation. The results presented in this manuscript revealed the molecular basis of Pan2-Pan3 interaction and are important contributions towards understanding the mechanism governing mRNA deadenylation by the Pan2-Pan3 complex. The manuscript is well written and is very pleasant to read. My only suggestion is that it might be worthwhile examining the effect of the Pan2 PID deletions on the deadenylation activity of Pan2-Pan3.

This is an excellent suggestion. We have performed new experiments to test the activities of Pan2–Pan3 complexes containing PID deletions. As shown in Figure 7, the deadenylation activity of *ct*Pan2–Pan3PKC decreases as more of the PID is removed. Complex formed with full length Pan2 removes a polyA tail after ~10 min. In comparison, complex formed with Pan2 Δ343-406 required ~90 min for complete deadenylation. We note that in these assays, *ct*Pan2–Pan3PKC first removes the polyA tail, but at later time points it degrades the 3' UTR. It is unclear why this occurs for *ct*Pan2–Pan3PKC but not for *sc*Pan2–Pan3ΔZnF.

Acceptance letter 03 May 2014

Thank you for submitting your final revised manuscript for our consideration. It has now been seen once more by one of the original referees (see comments below), and I am happy to inform you that there are no further objections, and that we have therefore now accepted the paper for publication in The EMBO Journal.

Thank you again for this contribution to The EMBO Journal and congratulations on a successful publication! Please consider us again in the future for your most exciting work.

Referee #2:

The authors have done a good job in addressing the comments raised in the previous round of review, making a strong manuscript even stronger. I am very enthusiastic regarding this submission.
